# 3D chromatin architecture, BRD4, and Mediator have distinct roles in regulating genome-wide transcriptional bursting and gene network

**DOI:** 10.1126/sciadv.adl4893

**Published:** 2024-08-09

**Authors:** Pawel Trzaskoma, SeolKyoung Jung, Aleksandra Pękowska, Christopher H. Bohrer, Xiang Wang, Faiza Naz, Stefania Dell’Orso, Wendy D. Dubois, Ana Olivera, Supriya V. Vartak, Yongbing Zhao, Subhashree Nayak, Andrew Overmiller, Maria I. Morasso, Vittorio Sartorelli, Daniel R. Larson, Carson C. Chow, Rafael Casellas, John J. O’Shea

**Affiliations:** ^1^ National Institute of Arthritis and Musculoskeletal and Skin Diseases, National Institutes of Health, Bethesda, MD, USA.; ^2^ Dioscuri Centre for Chromatin Biology and Epigenomics, Nencki Institute of Experimental Biology, Polish Academy of Sciences, 3 Pasteur Street, 02-093 Warsaw, Poland.; ^3^ National Cancer Institute, National Institutes of Health, Bethesda, MD, USA.; ^4^ National Institute of Allergy and Infectious Diseases, National Institutes of Health, Bethesda, MD, USA.; ^5^ National Institute of Diabetes and Digestive and Kidney Diseases, National Institutes of Health, Bethesda, MD, USA.

## Abstract

Discontinuous transcription is evolutionarily conserved and a fundamental feature of gene regulation; yet, the exact mechanisms underlying transcriptional bursting are unresolved. Analyses of bursting transcriptome-wide have focused on the role of cis-regulatory elements, but other factors that regulate this process remain elusive. We applied mathematical modeling to single-cell RNA sequencing data to infer bursting dynamics transcriptome-wide under multiple conditions to identify possible molecular mechanisms. We found that Mediator complex subunit 26 (MED26) primarily regulates frequency, MYC regulates burst size, while cohesin and Bromodomain-containing protein 4 (BRD4) can modulate both. Despite comparable effects on RNA levels among these perturbations, acute depletion of MED26 had the most profound impact on the entire gene regulatory network, acting downstream of chromatin spatial architecture and without affecting TATA box–binding protein (TBP) recruitment. These results indicate that later steps in the initiation of transcriptional bursts are primary nodes for integrating gene networks in single cells.

## Teaser

Perturbed scRNA-seq and mathematical modeling unveil role of transcriptional regulators in bursting and gene coexpression.

## INTRODUCTION

Prokaryote to human gene transcription occurs in bursts of RNA synthesis manifested by sporadic periods of gene activity punctuated by periods of apparent inactivity (*1*–*3*). Recognized since the 1970s as discontinuous transcription (*1*), transcriptional bursting is an important aspect of gene regulation although the precise mechanisms are largely obscured (*4*, *5*). This dynamic process reflects both upstream regulatory processes and the mechanisms of transcriptional activation and repression, which occur on timescales of seconds to days (*6*–*9*). Numerous investigations have delved into the mechanisms influencing transcriptional bursting. For example, steroids (*10*), local nucleosome rearrangements (*11*), and chromatin-modifying drugs altering chromatin dynamics (*12*) have been identified as regulators of burst frequency. Conversely, the levels of transcription factors (TFs) and MYC have been implicated in modulating burst size (*13*, *14*). Furthermore, certain regulators exhibit the capacity to influence both burst frequency and size (*15*–*17*). Yet, the larger implications for coordination of gene expression in single cells have not been studied.

Most of our understanding of transcriptional bursting comes from imaging in both fixed and living cells (*7*, *18*, *19*). Live-cell imaging provides real-time dynamics of transcription on all timescales (*7*), and smRNA-FISH (single-molecule RNA fluorescence in situ hybridization) studies have identified a role of enhancers and cohesin in modulating burst frequency (*20*–*23*). Even though these approaches have enabled tremendous progress, they can only be used to study selected genes at once. To overcome this limitation, a transcriptome-wide approach has recently been deployed to infer burst size and frequency by fitting a two-state mathematical model of transcription to single-cell RNA sequencing (scRNA-seq) data (*24*, *25*). This approach complements recent advances in large-scale single-molecule RNA FISH (*26*). scRNA-seq is more widely accessible and can examine transcripts and cell types that may not be accessible to imaging. Using this approach, Larsson *et al.* (*24*) proposed that enhancers encode bursting frequency, while TATA-containing promoters affect burst size. In addition, Ochiai *et al.* (*25*) showed that accumulation of transcription elongation factors correlates with frequency of bursting.

Thus far however, no transcriptome-wide study has attempted to integrate wide-ranging perturbations into a comprehensive framework for transcriptional activation that includes nuclear architecture, sequence-specific TFs, chromatin modifications, preinitiation complex (PIC) assembly, and elongation regulation. Doing so requires overcoming challenges to the mathematical modeling component. Quantifying parameter uncertainty is an area of active research in systems biology (*27*–*33*). Bayesian inference and model comparison are classic ways to address these problems but are nontrivial to implement and computationally expensive (*30*, *33*, *34*). Any method used to estimate parameters must be scalable to multiple datasets of tens of thousands of genes. Fast standard parameter optimization methods are reliant on initial starting points and can be confounded by local minima. Such approaches also do not adequately quantify the uncertainty of the estimated parameters or the suitability of the proposed models. Thus, it would be difficult to assess whether changes in parameters were due to the perturbation or simply that a wide range of parameters work equally well. Last, it is also not clear whether the prevailing two-state “telegraph” model is adequate to describe all the complexities of transcription. Rather, previous live-cell imaging has shown that more complicated models are often more appropriate (*7*).

Here, we develop an approach for fitting of single-cell sequencing data that, when coupled with experimental perturbations, reveals dynamic principles of transcriptional regulation. We developed a computationally efficient software suite called StochasticGene that performs Bayesian inference and analysis of arbitrary model architectures for a wide range of data types including scRNA-seq. The model produces posterior distributions of the parameters, which provides a quantification of the range over which parameters could vary without substantially affecting the fit to the data. We used this computational approach to interrogate the roles of key transcriptional regulators. That is, the MYC oncogene, cell type–specific enhancers, cohesin complex, CTCF (CCCTC-binding factor), BRD4 (Bromodomain-containing protein 4) and MED26 (Mediator subunit 26), which docks elongation factors (*35*). We find that these factors work through different kinetic mechanisms but that Mediator subunit 26 plays a unique role in coordinating gene expression in single cells.

## RESULTS

### Mathematical modeling infers transcriptional kinetics genome-wide

**Fig. 1.**
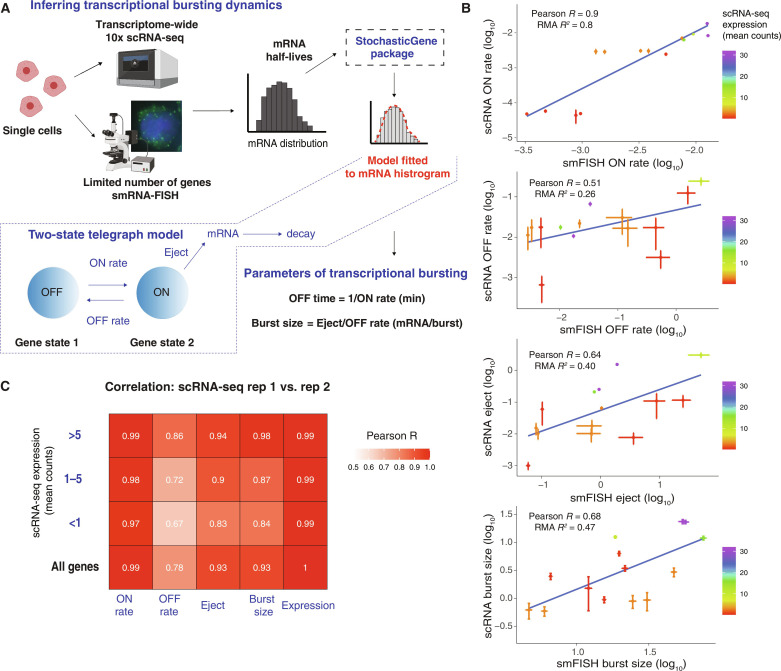
StochasticGene infers genome-wide rates of transcriptional kinetics. (**A**) Bayesian inference of the parameters of a two-state telegraph model of transcriptional bursting for scRNA-seq (10x Genomics) or smRNA-FISH data with mRNA half-lives as an input to StochasticGene. The two-state stochastic telegraph model is fitted to scRNA-seq or smRNA-FISH histograms for each gene. The model scheme is depicted in a box outlined by dashed lines and comprises transitions between an inactive OFF state and an active ON state, during which mRNA is emitted. There are four parameters: the ON rate, *k*_on_ (OFF to ON transition), OFF rate, *k*_off_ (ON to OFF transition), eject rate, *k*_eject_ (mRNA creation rate), and decay rate, *k*_decay_ (reflecting the mRNA disappearance rate). From these rates, we calculate the OFF time as 1/ *k*_on_ (in minutes) and the “burst size,” *k*_eject_/*k*_off_, which corresponds to the mean number of mRNA produced while in the ON state. (**B**) Correlation of inferred rates (log_10_) with scRNA-seq and smRNA-FISH (merged two smRNA biological replicates) in serum-activated HCT-116 cells. Pearson *R* and Reduced Major Axis (RMA) *R*^2^ as shown. Blue lines represent regression fit, *n* = 14. Error bars represent MAD (median absolute deviation), colored by mean scRNA-seq expression. (**C**) Correlation of rates (log_10_) inferred from two scRNA-seq biological replicates in serum-activated HCT-116 cells is grouped by mean expression. Pearson *R* as shown, *P* < 2.2 ×10^−16^. The sample sizes for different mean expression groups are as follows: *n* (rep1 and rep2 < 1 mean expr.) = 1250; *n* (1 < rep1 and rep2 < 5 mean expr.) = 4294; *n* (rep1 and rep2 > 5 mean expr.) = 2017; and *n* (all) = 7779. (A) created with BioRender.com.

Transcriptional bursting was modeled as a series of transitions between a finite number of states (*7*, *17*, *36*–*39*). One of the states is considered active or “ON” from which mRNA is emitted and then decays. The classic example is the two-state telegraph model (Fig. 1A), although bursting of some genes is better explained by models with more states (*7*, *39*). We used a Markov chain Monte Carlo (MCMC) algorithm to compute Bayesian posteriors of one-, two- and three-state models on scRNA-seq data acquired with a high-throughput platform capable of capturing thousands of cells (*40*). For stationary data, such as the scRNA distribution, no timescale can be specified, and rates are usually given with respect to the mRNA decay time. We specified the absolute timescale of the rates by fixing the mRNA decay time using mRNA half-lives measured in various cell lines, including HCT-116 (human colorectal carcinoma) and mouse natural killer cells, as well as mouse primary cells such as activated B, skin, and mast cells (fig. S1). We applied quality control filtering steps on the raw scRNA-seq data and computed the Bayesian posterior estimates of the model rates (fig. S2). Standard Bayesian model comparison measurements (e.g., Akaike Information Criterion and Watanabe-Akaike Information Criterion), which balance fit to data with the number of parameters, found that the two-state model was better for most genes compared to a one-state or three-state model given the data. In HCT-116 cells, none of the genes with the one-state model as a winner passed quality control. Only 0.78% (replicate 1, *n* = 18) and 1.57% (replicate 2, *n* = 29) had model 3 as the winner (data S1).

Consequently, we used a two-state model for all conditions, which has three free parameters (*k*_on_, *k*_off_, and *k*_eject_) and a fixed mRNA decay rate (*k*_decay_). Unless otherwise specified, we used the median of the Bayesian posterior to represent the rates. Often, *k*_off_ and *k*_eject_ are combined into a dimensionless number (*k*_eject_/*k*_off_), which can be construed as a burst size (number of mRNA produced while the gene is in the ON state). A major issue with fitting models to scRNA data is that not all mRNAs are captured (data S2 and S3). We addressed this deficiency by comparing the rates inferred from the scRNA-seq 10x platform to that inferred from smRNA-FISH and found that the most correlated measures between the two methods were *k*_on_ and burst size (*k*_eject_/*k*_off_) (Fig. 1B). We focused our analysis on the inverse of *k*_on_, which corresponds to the burst frequency or OFF duration (time between bursts in minutes), and the burst size (number of mRNA molecules produced per burst), which serves as a proxy value, as it depends on the yield of scRNA-seq. We also validated the rates by repeating experiments, which showed a high correlation between replicates (Fig. 1C) and cells in different stages of the cell cycle (fig. S3, A to D). Furthermore, to validate StochasticGene, we fit scRNA-seq data from Johnsson *et al.* (*41*) and compared our respective rate estimates. We observed a high correlation between *k*_on_ and burst size (fig. S4, A to C).

### Transcriptome-wide analysis reveals contrasting bursting kinetics of regulatory and housekeeping genes

**Fig. 2.**
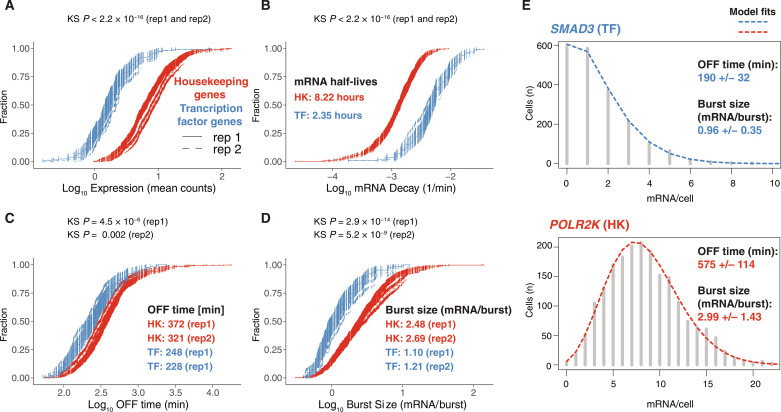
Regulatory genes burst more frequently than HK ones. (**A** to **D**) Comparison of HK (in red) and TF genes (in blue) in terms of (A) expression, (B) mRNA decay, (C) OFF duration, and (D) burst size in a steady-state HCT-116 cells [median values shown for each category, Kolmogorov–Smirnov (KS) test, *P* values as shown]. Two biological replicates are shown. The same decay rates, inferred from merged three biological replicates, were used for both scRNA-seq replicates; *n* (rep1: HK and TF) = 506 and 129; *n* (rep2: HK and TF) = 403 and 105 genes; 95% CI for the two replicates are shown. (**E**) Representative examples of TF and HK genes: scRNA-seq mRNA histograms with two-state telegraph model fits to scRNA-seq data (dashed lines) and, based on median posterior rates, inferred OFF time duration and burst size +/− estimation uncertainty based on MAD.

Prior work has revealed that housekeeping (HK) genes encode mRNA characterized by lower decay rates than mRNA of regulatory genes encoding TFs (*42*). However, it remained unclear whether high abundance of HK transcripts could be explained solely by their low decay. After confirming reproducibility of our method, we analyzed transcriptional bursting of HK and TF genes. For steady-state HCT-116 cells, we found that HK had higher expression (Fig. 2A) and produced mRNA with a lower decay rate compared to TF regulatory genes, with mRNA half-lives of 8.22 hours versus 2.35 hours, respectively (Fig. 2B). Our global analysis of scRNA-seq revealed that HK genes burst less frequently than TF genes (Fig. 2C). However, HK genes produced more mRNA per burst (Fig. 2D). Two representative examples are shown in Fig. 2E: a TF gene: *SMAD3*, a TF involved in transforming growth factor–β signaling versus a HK gene, *POLR2K*, which encodes one of RNA polymerase II (RNAP2) subunits; both exhibit the distinctive bursting behavior seen in their respective family of genes. Thus, HK genes exhibit lower mRNA decay rates and larger burst sizes despite a lower frequency of bursting compared to TF genes.

### MYC modulates burst size

**Fig. 3.**
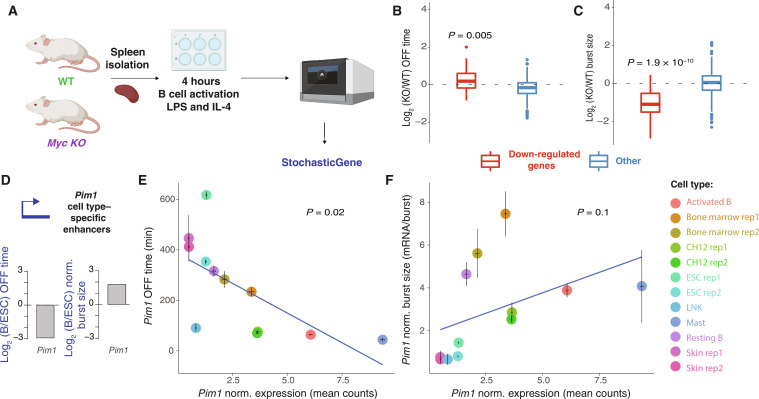
The impact of MYC and enhancers on bursting validate the transcriptome-wide approach. (**A** to **C**) Knockout (KO) of *Myc* affects burst size in splenic B cells: (A) Splenic B cells were isolated from wild-type (WT) and *Myc* KO mice and activated in vitro for 4 hours with lipopolysaccharide (LPS) and interleukin-4 (IL-4). Bursting kinetics was inferred by fitting a two-state telegraph model to scRNA-seq data. (B) Log_2_ fold change (LFC) of OFF time duration of down-regulated and other genes. (C) LFC of burst size of down-regulated and other genes (two-sided paired Wilcoxon test, *P* values as shown, *n* = 63 and 477, respectively). (**D**) LFC of (B/ESC) OFF time duration and normalized burst size of *Pim1* gene with cell type–specific set of enhancers in mouse ESC (average from two biological replicates) and activated B cells. (**E**) OFF time duration versus *Pim1-*normalized expression in different cell types, *n* = 12. (**F**) *N*ormalized burst size versus *Pim1*-normalized expression in different cell types, *n* = 12. Blue lines represent regression fit, and *P* values were obtained from the linear regression model. Error bars indicate the SEM for expression and uncertainty based on MAD for OFF time and burst size. All rates were inferred using the two-state model fitted to scRNA-seq data. (A) created with BioRender.com.

MYC is a canonical proto-oncogene that regulates many genes by functioning as a “global amplifier” of transcription (*43*, *44*). Recently, it was demonstrated with an optogenetic system and single RNA imaging that overexpression of MYC modulates burst duration and size, while not affecting burst frequency (*14*). Using activated primary *Myc*-deficient B cells (Fig. 3A) (*45*), we found smaller burst size and only a slight effect on OFF duration of down-regulated genes in MYC-depleted cells (Fig. 3, B and C), which validates that MYC modulates the amplitude rather than the frequency of transcription.

### Cell type–specific enhancers increase expression mostly through bursting frequency

Previously, it has been shown that distinct promoter-enhancer (P-E) contacts drive cell type–specific gene expression in pluripotent mouse embryonic stem cells (ESCs) and activated B cells (*46*). In contrast to globally acting MYC, we speculated, on the basis of imaging (*20*, *21*) and sequencing data (*24*), that enhancers gained during cell development should primarily drive the frequency of bursting. We thus compared bursting in ESCs and differentiated cells. One notable example was the *Pim1* gene, which showed differential enhancer usage in ESCs compared to B cells (*46*). This gene, regulated by the Janus kinase (JAK)–signal transducer and activator of transcription (STAT) (JAK/STAT) signaling pathway, encodes a Ser/Thr protein kinase (*47*) and has higher expression in activated B cells compared to ESCs. We found that higher *Pim1* expression can be explained by shorter OFF duration (log_2_FC = −2.92) and a smaller increase in normalized burst size (log_2_FC = 1.78) (Fig. 3D). To examine the relation of *Pim1* expression with OFF time and burst size across various cell types, in addition to mouse ESCs and activated B cells, we performed normalization of expression and burst size. This normalization accounts for differences in sequencing depth across cell types, which affect the yield and, consequently, the eject rate used to compute burst size (fig. S5A). We examined primary B220^+^ bone marrow cells, splenic resting and activated B cells, ES cells, mast cells, skin cells, immortalized natural killer cells, and the mouse B cell tumor CH12 cells (*n* = 8 cell types). We conducted regression analyses and found that *Pim1* expression across cell types significantly explains the variability of OFF time (*P* = 0.02), with burst size showing a weaker association (*P* = 0.1) (Fig. 3, E and F).

To further investigate how cell type–specific enhancers regulate transcriptional bursting, we analyzed the scRNA-seq data for other chromatin interaction analysis with paired-end tags (ChIA-PET)–identified ESC and B cell type–specific enhancers (*46*) (fig. S5, B to D). We compared fold change (FC)–normalized expression (B cells/ESCs), OFF duration, and normalized burst size in both genes that gained enhancer activity and genes that lost enhancer activity during development (*46*). Gained enhancers were observed in B cells but were absent in ESCs, while lost ones were present only in ESCs. After filtering, we retained 29 genes that gained two or more enhancers during development and 62 genes that lost two or more cell-specific enhancers. As expected, genes that gained enhancers showed higher expression in activated B cells. They had shorter OFF duration and to a lesser extent larger burst sizes (fig. S5C). When we applied a more stringent threshold (four or more enhancers gained or lost), we found that B cell type–specific genes linked to B cell enhancers were characterized by shorter OFF duration, but the burst size was not significantly different from genes with lost enhancers (fig. S5D). Thus, the accumulation of cell-specific enhancers during development correlates with a decrease in transcription OFF duration. Together, our study of the role of MYC and enhancers in transcriptional bursting validates our transcriptome-wide approach as a tool that can be applied to perturbed systems.

### The perturbations of cohesin, BRD4, and MED26 have distinct effects on transcriptional bursting

**Fig. 4.**
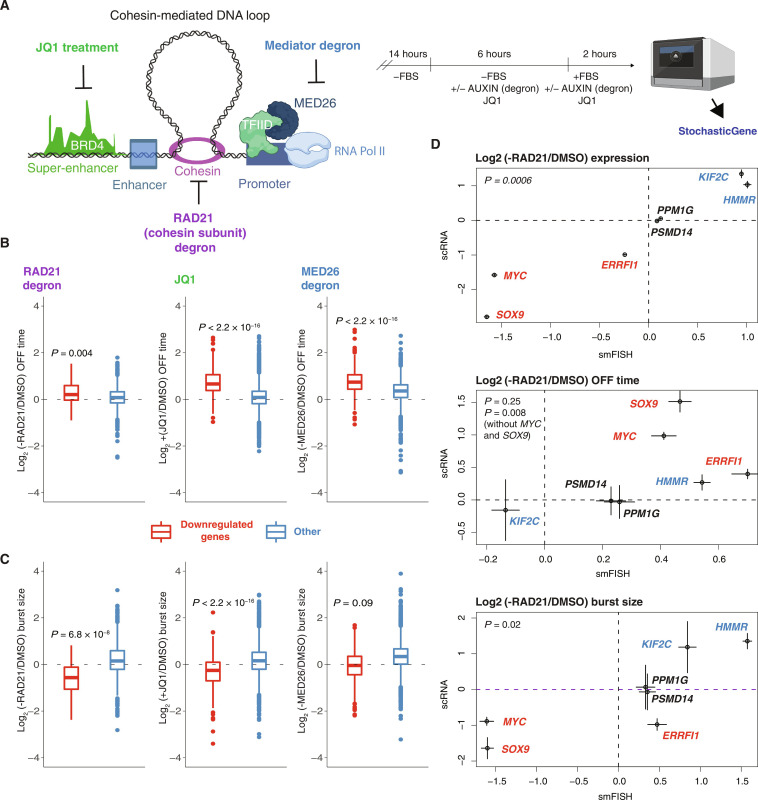
Cohesin and BRD4 regulate the burst size and OFF duration, while MED26 affects the frequency. (**A**) On the left is a scheme illustrating key transcription regulators that were perturbed: BRD4 (JQ1 treatment), cohesin complex [RAD21 degron (*54*)], and Mediator subunit interacting with TFIID (*35*): MED26 [MED26 degron (*55*)]. On the right is a scheme outlining the experimental design: HCT-116 cells were starved for 14 hours and then treated with auxin (RAD21 and MED26 degron) or 500 nM JQ1 for 6 hours in the absence of fetal bovine serum (FBS). Two hours after the addition of serum in the presence of auxin or JQ1, cells were captured, and scRNA-seq was performed. A two-state telegraph model was fitted to the scRNA-seq data to infer parameters of bursting in control and treated cells using the same decay rates for both conditions. (**B**) LFC of OFF time duration of significantly down-regulated genes (in red) upon RAD21 loss (*n* = 70), JQ1 treatment (*n* = 397), MED26 loss (*n* = 729), and other genes (in blue). Two-sided paired Wilcoxon test, *P* values as shown. (**C**) LFC of burst size of significantly down-regulated and other genes. (**D**) Comparison of LFC (RAD21/DMSO) expression (top), OFF time (middle), and burst size (bottom) inferred using scRNA (scRNA-seq) and smFISH (smRNA-FISH: merged two biological replicates) data. Red, significantly down-regulated genes; black, unaffected genes; blue, significantly up-regulated genes. *P* values from regression: log(scRNA) ~ log(smFISH); Error bars indicate the SEM for expression and uncertainty based on MAD for OFF time and burst size (*n* = 7 genes). (A) created with BioRender.com.

Cohesin, bromodomain-containing protein BRD4, and Mediator exert critical and distinct functions in transcriptional regulation. The cohesin complex plays a key role in three-dimensional (3D) chromatin architecture by facilitating the formation of topologically associating domains through loop extrusion (*48*–*50*), which is halted by CTCF (*51*, *52*). Many down-regulated genes upon cohesin loss are located near super-enhancers (SEs) (*52*) that colocalize with Mediator and BRD4 (*53*). To investigate the effects of these perturbations on transcriptional kinetics while avoiding secondary effects, we used an auxin-induced degron system to deplete the cohesin subunit RAD21 (*54*) and MED26 (*55*). In addition, to displace transcriptional coactivator BRD4 (*56*), we exposed cells to JQ1 (500 nM) (fig. S6, A and B). All treatments were conducted under the same conditions: HCT-116 cells were starved and serum activated for 2 hours before harvesting (Fig. 4A). Using bulk mRNA-seq, we observed a similar impact of these perturbations on RNA levels of significantly down-regulated genes, a category that was applied to the following analyses of transcriptional kinetics (fig. S7).

Our transcriptome-wide analysis revealed that down-regulated genes (fig. S7, A to D) exhibited significantly increased OFF duration for all three perturbations [log_2_FC = 0.2 (RAD21 degron), 0.66 (JQ1 treatment), and 0.74 (MED26 degron)] (Fig. 4B), but the effect on burst size was not universal. RAD21 loss induced the largest effect on burst size for down-regulated genes (log_2_FC = −0.57), JQ1 had a weaker impact (log_2_FC = −0.25), and MED26 loss had no significant impact on median burst size (Fig. 4C). Conversely, other genes showed almost no changes in parameters upon RAD21 loss and JQ1 treatment. On the other hand, upon MED26 loss, these genes had bigger burst sizes with longer OFF duration, suggesting compensatory mechanisms (*57*) (Fig. 4, B and C). To evaluate the global impact of MED26 depletion on the transcriptome, we performed spike-in normalized mRNA-seq and observed a global but mild down-regulation, consistent with previous reports (*57*, *58*) (fig. S8, A and B). This control indicates that the compensatory mechanism is only partial.

To eliminate the possibility that differences in mRNA decay induced by the treatments could affect the interpretation of our results, we conducted RNA-seq following these perturbations and transcription inhibition with actinomycin D to determine mRNA half-lives (fig. S9). However, it is important to note that, despite incorporating spike-ins as a control to address technical noise, not only the correlation may be relatively low but also many values could potentially shift between replicates (*59*). This introduces additional noise into the bursting analysis. We found that the correlation of mRNA half-lives in control and perturbed samples was comparable to that between replicates under perturbations (fig. S9, A and B). Nevertheless, to ensure that changes in half-lives did not affect the inferred rates, we refitted the data using half-lives measured under control and perturbations (fig. S9, C and D). Consistently, we observed that all perturbations similarly affected the OFF time and burst size of down-regulated genes when applying the same decay rates to both control and perturbation conditions (Fig. 4, B and C). Similarly, for other genes, bursting parameters remained almost unchanged upon RAD21 loss. The OFF time was longer and burst size bigger upon MED26 loss; however, the OFF time was also longer under JQ1 treatment (fig. S9, C and D), but we cannot rule out the possibility that it might be the effect of noise associated with mRNA decay interference. Together, we found that although all three perturbations affected OFF duration (i.e., burst frequency), they had different impacts on burst size.

To assess the distinct impact of cohesin loss on burst size, we used complementary approaches and compared smRNA-FISH and scRNA-seq inferred bursting parameters in seven genes following cohesin perturbation. To evaluate the direction and magnitude of FCs [−RAD21/dimethyl sulfoxide (DMSO)] of parameters from the two methods, we performed linear regression (Fig. 4D). We analyzed two genes that, based on bulk mRNA-seq, remained unaffected by this perturbation (*PPM1G* and *PSMD14*), three down-regulated genes (*SOX9*, *MYC*, and *ERRFI1*), and two up-regulated genes (*HMMR* and *KIF2C*). Both methods exhibited good agreement for expression changes. While we observed discrepancies in the magnitude of OFF time changes for two down-regulated genes (*MYC* and *SOX9*), the directions of changes for these gene were in agreement between the two methods. For one down-regulated gene, *ERRFI1*, despite consistently showing longer OFF times, we observed an inconsistency in burst size, possibly due to a disparity in down-regulation magnitude between the two methods. However, despite this inconsistency, we also observed good agreement for burst size FCs inferred from smRNA-FISH and scRNA-seq. Together, this comparison confirmed the impact of cohesin loss on OFF time and burst size (Fig. 4D). We also analyzed bursting kinetics upon cohesin loss 30 min after release from fetal bovine serum (FBS) starvation, and in serum-starved HCT-116 cells. In both cases, we observed a higher correlation of expression change (−RAD21/DMSO) with burst size change (fig. S10, A and B). To evaluate whether the results observed in HCT-116 cells could be generalized to other cell types, we induced auxin-mediated RAD21 degradation in mouse ESCs (*60*) and confirmed that transcriptional output of this perturbation is dictated mostly by change in burst size (fig. S10C). To further investigate how genome architecture affects transcriptional bursting, we depleted CTCF with an auxin-based approach in ESCs (*61*) (fig. S10D). Although burst size was still the most affected parameter, we observed a bigger impact of CTCF removal on OFF duration (fig. S10, C and D). Together, we observed that factors shaping the 3D chromatin architecture and BRD4 regulated bursting frequency and burst size of down-regulated genes, whereas MED26 modulated bursting frequency.

### MED26 has the most profound impact on the gene network

**Fig. 5.**
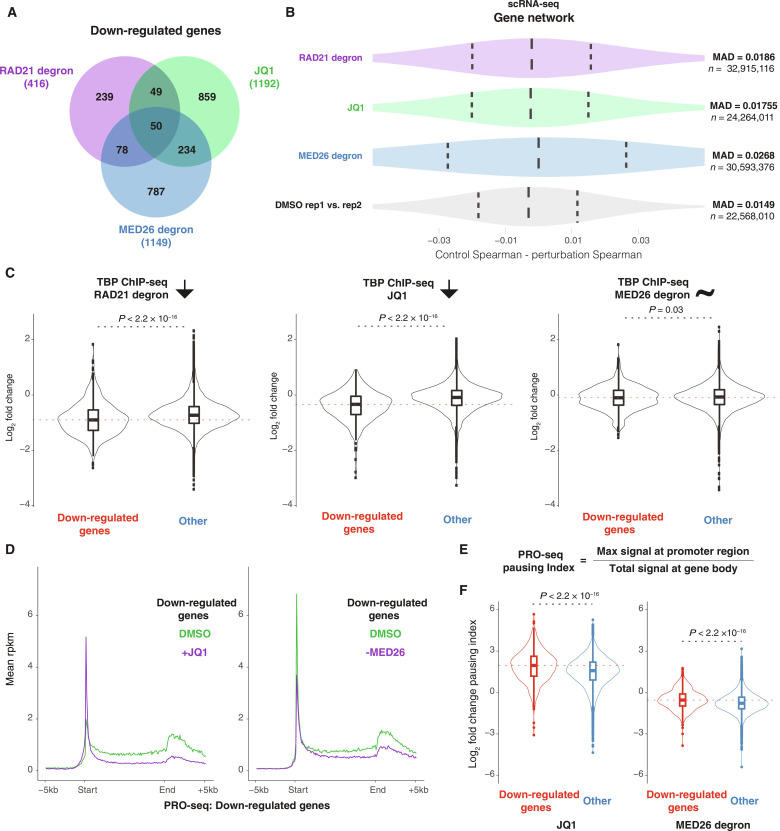
Cohesin, BRD4, and MED26 play distinct role in transcription and gene network regulation. (**A**) Venn diagram of down-regulated genes in RAD21 degron, JQ1-treated, and MED26 degron HCT-116 cells. (**B**) Impact of tested perturbations on gene-gene correlations across single cells based on scRNA-seq (MAD, *n* represents number of gene pairs). (**C**) Impact of tested perturbations on TBP binding based on ChIP-seq [*n* of peaks: RAD21: 644 (down) and 18,837 (other); JQ1: 400 (down) and 8148 (other); MED26: 828 (down) and 13,104 (other), two-sided unpaired Wilcoxon test, *P* values as shown]. (**D**) PRO-seq composites of transcriptionally engaged RNA polymerases upon JQ1 treatment (left: *n* = 425) and MED26 perturbations (right: *n* = 475) at down-regulated genes; control (DMSO) in green and perturbation in magenta. (**E**) Formula for PRO-seq pausing index. (**F**) LFC pausing index upon JQ1 treatment (left violin plot) and MED26 perturbation (right violin plot); two-sided unpaired Wilcoxon test, with *P* values as shown. Down-regulated genes are represented in red (*n* = 1017 for JQ1 and 1027 for MED26 loss), while other genes are shown in blue (*n* = 11,235 for JQ1 and 11,407 for MED26 loss).

The preceding analyses treat all genes independently and individually, but scRNA-seq is inherently multidimensional. Intrigued by the debate surrounding the association between cohesin, BRD4, and Mediator (*52*, *58*, *62*–*64*), we further investigated down-regulated genes following these perturbations and found that only 50 genes were commonly affected by these perturbations suggesting a distinct mechanism of action (Fig. 5A and data S4 to S7). Hence, we aimed to investigate whether MED26, given its distinct influence on bursting, exerts varying effects on the gene network. We hypothesized that changes in transcriptional bursting in individual cells would affect the coordination of expression between genes. We measured the underlying gene network by calculating the correlations between pairs of genes at the single-cell level. Here, when we refer to the gene network, we are referring to the underlying processes that dictate the correlated expression of pairs of genes in individual cells. To quantify the effect of a specific perturbation, we quantified the change in the correlation coefficient with each perturbation and compared the change to a control (the change in correlation for each pair genes in replicates). Using this approach, we found a consistent spread for correlation changes and with a similar amount of variability except for the MED26 perturbation, which had higher variability in terms of absolute correlation coefficient changes (Fig. 5B). To highlight this result, we show the median absolute deviation (MAD) of each distribution in Fig. 5B and further show that the results are consistent across multiple repeats in fig. S11 (A and B). We confirmed that the distinctiveness of the MED26 perturbation was not due to differences in gene expression levels (fig. S11, C and D). Overall, the analysis suggests that MED26 plays a more profound role in dictating the coexpression of genes at the single-cell level when compared to the other factors.

### MED26 regulates gene network via BRD4 and RNAP2 pause release

On the basis of our findings regarding MED26, we attempted to elucidate how this factor affects transcription at a stage distinct from other examined regulators. We focused on TBP (TATA box–binding protein), a component of the transcription PIC (*65*). We found that RAD21 and BRD4 act upstream of the PIC formation, as indicated by lower TBP binding at promoters of down-regulated genes (Fig. 5C). In contrast, we did not observe notable differences of TBP binding at promoters of genes down-regulated by acute MED26 loss compared to other genes. MED14 has been shown to act downstream of the spatial chromatin structure driven by cohesin (*58*). Consistent with this finding, we observed minimal impact on RAD21 binding upon MED26 loss (fig. S12A), implying that MED26 also functions downstream of the 3D chromatin structure.

Subsequently, we performed RNAP2 chromatin immunoprecipitation sequencing (ChIP-seq) under cohesin, BRD4 (JQ1 treatment) and MED26 perturbations. Our analyses of bursting revealed that under all three perturbations, there was an increase in the OFF duration between transcriptional bursts. However, it was only MED26 that specifically affected the OFF duration of the bursts without altering the median burst size of down-regulated genes (Fig. 4, B and C). When comparing RNAP2 binding at down-regulated genes following MED26 loss, we observed reduced RNAP2 occupancy at the transcription start site but negligible changes in the gene body compared to other perturbations (fig. S13). This suggests that RNAP2 recruitment is likely affected by the degradation of MED26. We also observed that the RNAP2 signal varies under DMSO treatment at genes affected by all three perturbations (fig. S13). This finding indicates that the regulation of RNAP2 initiation and proximal pause release is controlled in a gene-specific manner by cohesin, BRD4, and MED26 (fig. S13). In addition to RNAP2 ChIP-seq, to assess the impact of MED26 perturbation on transcriptionally engaged RNA polymerases, we performed precision nuclear run-on sequencing (PRO-seq) (*66*). As a control, we also conducted PRO-seq upon JQ1 treatment and found that both treatments decreased levels of RNA polymerases at the gene body and promoter region in the case of MED26 loss (Fig. 5D). These findings further support the role of MED26 as a regulatory switch for transcription.

To explore potential downstream mechanisms following PIC formation, we next turned to BRD4, which is known to coordinate elongation (*67*). We hypothesized that MED26 could tune bursting kinetics by interacting with BRD4 at the promoter region. We found affected BRD4 binding at promoter regions upon acute loss of this Mediator subunit (fig. S12B). Consistently, we observed changed BRD4 occupancy at promoters of down-regulated genes compared to up-regulated ones following perturbations (fig. S12C).

Last, using PRO-seq data, we computed a pausing index, which is the ratio of promoter-proximal to gene-body RNA polymerase density (*68*) (Fig. 5E). We observed that down-regulated genes exhibited increased FCs (JQ1/DMSO) in pausing compared to other genes, consistent with previous studies (*69*) (Fig. 5F). Similarly, we observed increased pausing indices of down-regulated genes relative to other genes upon MED26 depletion (Fig. 5F). However, we noted that the FCs (−MED26/DMSO) of pausing indices had lower values than after JQ1 treatment. This supports our RNAP2 ChIP-seq findings, which revealed affected binding of RNA polymerases upon perturbation of this subunit (Fig. 5F and fig. S13).

Our results highlight the distinct roles of cohesin, BRD4, and MED26 in modulating transcriptional bursting. Moreover, we demonstrate that these modulators act at different stages of transcription, with MED26 exerting a higher impact on gene network regulation. Our findings also suggest that MED26, in conjunction with BRD4, acts as a binary ON/OFF switch of transcription.

## DISCUSSION

scRNA-seq combined with computational approaches provides a useful tool to investigate the heterogeneity of transcription across thousands of genes. These distributions can be used to infer dynamic regulation. In contrast, imaging of mRNA in live or fixed cells can only be used to study selected genes. We used our transcriptome-wide approach to investigate the kinetics of transcription across the genome under a wide range of conditions to learn their impact on transcriptional bursting and gene coexpression at the single-cell level.

One limitation of using smRNA-FISH or scRNA-seq as input for a mathematical model of transcription is the challenge in accurately capturing the timescale for the steady-state mRNA distributions obtained with both methods. However, smRNA-FISH has been applied to capture dynamic changes over time in response to various stimuli, such as serum induction (*16*) or cytokine stimulation (*37*). To address this limitation, we measured genome-wide mRNA half-lives for several cell types and used this information to estimate the OFF duration. Another limitation is the loss of RNA because scRNA-seq only captures a fraction of the mRNA (data S2). Moreover, although adequate given the resolution of scRNA-seq, the telegraph model is known to be a simplification of transcription dynamics. While live-cell imaging shows that three gene states (one active and two inactive states) are usually justified in fitting the data, this is rarely the case for scRNA data (data S1), because of the lower yield. As a result, there are fewer parameters in the two-state model used in this paper compared to the models used in the live imaging studies (*7*, *39*). This ambiguity obscures the relationship between the parameters for different models as the parameters of the smaller model will be amalgamations of the parameters of the larger model. Therefore, bursting parameters inferred using the two-state model can differ from live imaging using the MS2/PP7 system or smRNA-FISH. Our data indicated that the OFF time, in steady-state HCT116 cells, for the presented genes lasts hours (Fig. 2), and the median OFF time for all genes was about 6 hours. These times are in agreement with other studies using scRNA-seq to infer the frequency of bursting, which showed that the time between bursts can last 4 to 6 hours (*24*, *41*). This is also in line with imaging data of thousands of genomic loci that revealed that OFF time lasts 2 to 4 hours (*15*) and live imaging using the MS2/PP7 system that reveals that the *TFF1* gene can be inactive for more than 10 hours (*7*). Modeling of this data predicts that *TFF1* can be inactive on the timescale of days (*7*). For an additional 10 genes, live-cell imaging using the MS2/PP7 systems showed that the time of inactivity is gene dependent and lasts up to ~90 min (*39*). Nevertheless, the limitations of scRNA-seq may result in some discrepancies between high-resolution single-gene imaging (smRNA-FISH and live imaging using the MS2/PP7 system) and high-throughput inference of bursting parameters using data with lower resolution, like scRNA-seq. To compensate for this bias, we compared scRNA-seq to the gold-standard method: smRNA-FISH. It can be shown theoretically that if the fraction captured by scRNA is completely random, then only the eject rate will be affected (see Materials and Methods). We validated that rate parameters inferred from scRNA-seq were correlated with those from smRNA-FISH and thus scRNA-seq can provide reliable estimates of OFF duration and burst size in perturbed samples. Genes with an insufficient number of mRNA counts were removed during our quality control filtering.

In this study, we demonstrated the utility of our approach in comparing the transcriptional kinetics of genes with different functions. HK genes are often referred to as constitutive genes (*70*, *71*), but it remains unclear whether the high abundance of HK transcripts depends solely on their long mRNA half-lives, as previously shown (*42*), or on their distinct transcriptional kinetics. If HK genes were transcribed constitutively, we might expect a higher bursting frequency of HK genes compared to TF genes. However, contrary to this expectation, we observed that mRNA half-lives and, unexpectedly, burst size mainly determine the high expression of HK genes, rather than frequency. Our model indicates that their OFF time is even longer than that of TF genes. Therefore, HK genes, by producing mRNA with low decay, may not require such frequent bursting. We also note that our analysis of these gene parameters was conducted in steady-state cells, and it is possible that regulatory TF genes, which have a smaller burst size than HK genes, could increase burst size upon cell activation. Gene expression can be enhanced upon stimulation through an even higher frequency of bursting and, additionally, by an increase in burst size. It has been demonstrated that TF concentration could modulate the frequency of bursting. Further increases in expression could occur through the modulation of TF binding duration and, consequently, burst duration (*16*).

Our genome-wide approach also allowed us to identify regulators of transcriptional bursting. We focused on the effect of cohesin, BRD4, and the Mediator complex subunit MED26 in serum-activated HCT-116 cells. All perturbations affected frequency of bursting, whereas cohesin loss and BRD4 perturbation also had an impact on burst size. Previous studies based on imaging of selected genes have revealed that JQ1 treatment can affect both the frequency and size of bursting depending on the gene in murine G1E cells (*17*). Our result is also consistent with previous findings in murine activated macrophages and steady-state HCT-116 cells that show that cohesin regulates frequency (*22*, *23*). The finding that smRNA-FISH spot intensities (*22*) or volumes (*23*) were almost unchanged upon cohesin loss in those experiments does not contradict our finding that burst size was decreased because the fluorescence intensity is a measure of nascent RNA density, while our burst size measures the number of mRNA molecules produced per burst and thus carries information about the duration of the transcriptional event. The effect on burst size along with OFF duration across down-regulated genes upon cohesin loss suggests that this complex might also play a role other than bringing together promoters and enhancers. If cohesin exclusively affected P-E contacts, then it would only affect OFF duration and not burst size because enhancers primarily modulate OFF duration [Fig. 3 and (*21*, *24*, *72*)]. This agrees with a recent study (*73*), which finds a weak impact of cohesin depletion on P-E contacts. By contrast, we found that MYC governs burst size across the genome in murine activated B cells, in agreement with imaging in live human cells (*14*).

The role of the Mediator complex in bursting had not been studied on a genome-wide scale until now. It was not clear whether Mediator regulates burst size or the frequency of bursting. We used a transcriptome-wide approach and found that genes down-regulated by MED26 loss exhibited a lower frequency of bursting, but the median FC of burst size was not affected. Unexpectedly, we also found that genes not significantly down-regulated upon MED26 loss had lower frequency and bigger burst size. Our spike-in normalized bulk mRNA-seq proved that even acute MED26 loss induced a global but mild down-regulation of expression, in accordance with previous results (*57*, *58*). The increased burst size might partially compensate for the longer OFF time. A compensatory mechanism upon Mediator loss was previously observed by Jaeger *et al.* (*57*). In their study, the authors perturbed the MED14 subunit of the Mediator complex, affecting the initiation of transcription, and observed a compensatory mechanism through the activation of positive transcription elongation factor (P-TEFb) (*57*). We also observed a slight increase in RNAP2 binding at the gene body of genes other than those down-regulated, specifically after MED26 depletion but not JQ1 treatment (fig. S14, A and B). Notably, there was a greater increase in burst size upon MED26 loss compared to the effects of other treatments (Fig. 4D), suggesting a compensatory mechanism in response to MED26 perturbation through an increase in burst size and RNAP2 binding at the gene body. However, we acknowledge that this increase is subtle. Consequently, this mechanism may only partially compensate for MED26 loss, as we observed a mild but global down-regulation when analyzing spike-in normalized bulk mRNA-seq (fig. S8B). Because MED26 was shown to interact with P-TEFb (*35*), the mechanism upon MED26 loss might differ from MED14 perturbation or may be gene dependent. Together, the possible compensatory mechanism requires further investigation to be fully understood.

While much is gained in analyzing single genes in isolation, scRNA-seq provides the ability to examine correlations between genes in single cells, which could help to explain how cells maintain cellular identity through transcriptional regulation. Several factors could obscure the significance of these correlation changes (*74*), including RNA detection noise, RNA persistence from preperturbation exposure, and variations in RNA degradation. Given this context, our analysis revealed that JQ1 treatment and perturbations in 3D chromatin architecture only had weak effects on the correlation between expressed genes. However, MED26 depletion had a greater effect on gene-gene correlation along with distinct effects on bursting. Although the network analysis was performed separately from the bursting, we speculate that changes in transcriptional kinetics, particularly an increase in OFF time resulting in a significant down-regulation of affected genes, can influence the temporal expression of other genes. The basis of this proposal is that this influence may occur through cobursting—a phenomenon where genes tend to burst together more often if they are localized closer in spatial, though not necessarily genomic, scale (*74*). However, the phenomenon of cobursting is not fully understood. For instance, it has been demonstrated that glucocorticoids can activate not only proximal but also distal genes through cobursting (*75*), potentially influencing cellular identity and the gene network. Therefore, MED26 depletion could affect the gene network by increasing the OFF time of specific genes and, consequently, perturbing the coexpression of specific gene pairs.

In search for a molecular mechanism and the reason for a distinct role of MED26 in regulating transcriptional bursting and gene coexpression, we performed ChIP-seq for TBP, which is involved in the formation of the transcription PIC (*76*). We found that although loss of cohesin and perturbation of BRD4 affected TBP binding genome-wide, MED26 did not. Also, Takahashi *et al.* (*35*) showed that MED26 knockdown has no major effect on TBP occupancy at *c-MYC* and *HSP70* promoters. Together, it suggests that MED26 acts downstream of TBP recruitment. To explore potential mechanisms, we turned our attention to BRD4, which has been shown to coordinate recruitment of pause release factor (*67*). Our genome-wide analysis revealed that MED26 depletion affects BRD4 binding at promoters to a greater extent than SEs, in contrast to the effects of JQ1 treatment. In addition, we observed altered BRD4 binding at promoters of differentially expressed genes upon MED26 loss. These findings suggest that MED26 regulates the release of stalled RNAP2, possibly through its interaction with BRD4. Our PRO-seq analysis revealed that genes down-regulated by both JQ1 treatment and MED26 perturbations have higher RNA polymerase pausing indices compared to other genes. However, we observed that MED26 perturbation likely affects RNAP2 binding, as reflected in RNAP2 ChIP-seq and the negative values of log_2_ FC of pausing indices upon MED26 loss. Notably, MED26 has been demonstrated to interact with RNAP2 (*35*). In addition, other studies have shown interactions between certain subunits of the Mediator complex and BRD4 (*77*–*81*). However, it remains to be elucidated whether the interaction between MED26 and BRD4 is direct or indirect. Previously, it had been also found that MED26 can act as a molecular switch docking first transcription initiation factors, and then exchange them for complexes involved in elongation, such as P-TEFb (*35*). Together, our data suggest that MED26 is part of a rate-limiting step in transcriptional bursting, likely working through the binding and release of RNAP2, and this late step in the transcription cycle is a critical factor in regulating the gene network.

Overall, our findings underscore the intricacy of transcriptional regulation, enhancing our understanding of how cellular identity is maintained through complex transcriptional networks. Applying the proposed strategy to other perturbations could shed light on the robustness of the network, providing a deeper insight into cellular strategies that maintain gene expression homeostasis by modulating transcriptional bursting dynamics. Combining rapidly evolving single-cell imaging and sequencing methods (*82*) alongside microscopy super-resolution techniques and innovating new computational tools (*7*, *83*, *84*) will further enhance our ability to unravel the complexities of transcriptional regulation.

## MATERIALS AND METHODS

### Cell culture

HCT-116 degron cell lines: RAD21- mAID-mClover (*54*) and HCT116-OsTIR1-MED26-AID (*55*) were cultured in McCoy’s 5A medium supplemented with l-glutamine (Gibco), 10% FBS (Gemini), penicillin (100 U/ml), and streptomycin (100 μg/ml) (Gibco), at 37°C and 5% CO_2_. Starvation: Cells were FBS depleted for 14 hours, followed by an additional incubation with FBS-free medium for another 6 hours in the presence of DMSO (control), 500 μM auxin [indole-3-acetic acid (IAA)] (Sigma-Aldrich) for degradation of the auxin-inducible degron (AID)–tagged RAD21 and MED26 or 500 nM JQ1 (Sigma-Aldrich) for BRD4 displacement. Cells were analyzed after addition of FBS in the presence of DMSO or drugs at time points: 0 hours (RAD21 degron), 0.5 hour (RAD21 degron), and 2 hours (RAD21 and MED26 degron, JQ1 treatment).

ES (E14) RAD21 (*60*) and CTCF degron (*61*) cell lines were cultured in knockout (KO) Dulbecco’s modified Eagle’s medium (Gibco), ES cell–qualified FBS (American Type Culture Collection), 1:100 Glutamax (Gibco), 1:100 sodium pyruvate (Gibco), 1:100 nonessential amino acids (NEAA) (Gibco), penicillin (100 U/ml) and streptomycin (100 μg/ml) (Gibco), 1:1000 2-mercaptoethanol (Gibco), and 1:10,000 leukemia inhibitory factor (10,000×) on 0.1% gelatin-coated dishes at 37°C; 5% CO_2._ For the AID-tagged proteins, 500 μM auxin (IAA) (Sigma-Aldrich) was added for 6 hours (RAD21) or 24 hours (CTCF). CH12 B lymphoma cells were cultured in RPMI 1640 media (Gibco) supplemented with 10% FBS (Gemini), penicillin (100 U/ml) and streptomycin (100 μg/ml; Gibco), 1:1000 2-mercaptoethanol (Gibco), at 37°C; 5% CO_2._ NK (cell line) cells were cultured in RPMI 1640 media (Gibco) supplemented with 10% FBS (Gemini), 1:100 Glutamax (Gibco), penicillin (100 U/ml) and streptomycin (100 μg/ml; Gibco), 10 mM Hepes, 1:100 NEAA (Gibco), 1:100 sodium pyruvate (Gibco), 1:1000 2-mercaptoethanol (Gibco), and interleukin-2 (IL-2) (200 IU/ml; Sigma-Aldrich).

Primary cells were derived from male and female mice of strains as follows: Primary wild-type (WT) and *Myc* KO B cells were derived from *Myc* flox/flox mice (WT) or RosaCreERTam/*Myc* flox/flox mice (KO) treated with tamoxifen (Sigma-Aldrich) (three times of intraperitoneal 1 mg per mouse at 4, 3, and 1 day before sacrifice). The deletion of floxed allele was checked as previously (*45*) with polymerase chain reaction or intracellular staining of MYC protein. B cells were isolated from spleen of WT and KO mice, enriched with EasySep Mouse B Cell Isolation KIT (STEMCELL Technologies) and activated for 4 hours with lipopolysaccharide (LPS) (50 mg/ml) and IL-4 (2.5 ng/ml) in RPMI 1640 medium (Gibco) supplemented with 10% FBS (Gemini), 1:100 Glutamax (Gibco), 1:100 sodium pyruvate (Gibco), 1:100 NEAA (Gibco), penicillin (100 U/ml) and streptomycin (100 μg/ml; Gibco), 1:1000 2-mercaptoethanol (Gibco) and Hepes (Gibco), at 37°C and 5% CO_2_. B220-positive primary bone marrow B cells were derived from C57BL/6 J WT mice, enriched with the CD45R (B220) MicroBeads Kit (Miltenyi Biotec) and the Dead Cell Removal Kit (Miltenyi Biotec).

Splenic B cells were derived from C57BL/6 J WT mice immunized with a keyhole limpet hemocyanin (KLH; 25 μg per footpad, Sigma-Aldrich) in the presence of 10% Freund’s complete adjuvant (Sigma-Aldrich). After 5 days, B cells were isolated from spleen, enriched with the EasySep Mouse B Cell Isolation KIT (STEMCELL Technologies), and activated for 72 hours with LPS (50 mg/ml), IL-4 (2.5 ng/ml), and anti-CD180 in RPMI 1640 medium (Gibco) supplemented with 10% FBS (Gemini), 1:100 Glutamax (Gibco), 1:100 sodium pyruvate (Gibco), 1:100 NEAA (Gibco), penicillin (100 U/ml) and streptomycin (100 μg/ml; Gibco), 1:1000 2-mercaptoethanol (Gibco), and Hepes (Gibco), at 37°C and 5% CO_2._ To measure mRNA half-lives in activated B cells, actinomycin D was added after 2 days of stimulation with LPS, IL-4, and anti-CD180 (more details: mRNA half-lives estimation).

Skin cells for scRNA-seq: whole epidermis from mouse neonates. Keratinocytes were isolated from Fvb neonate (P0-P2) skin (*n* = 2) as described by Lichti *et al.* (*85*). Cells were subjected to the MACS Dead Cell Removal Kit (Miltenyi Biotech) using the manufacturer’s protocol. Skin cells for mRNA half-lives estimation: primary keratinocyte. Skins from a pool of Fvb neonatal mice were collected at P0-P2 and incubated overnight in Dispase II (2.0 U/ml; Sigma-Aldrich) in KBM-Gold (Lonza) on an end-over-end rotator. The following day, skins were washed twice in 1× phosphate-buffered saline (PBS) (Sigma-Aldrich), and epidermises were separated from dermises. Epidermises were placed basal side down and floated in prewarmed 0.25% trypsin-EDTA (Thermo Fisher Scientific) for 20 min on an orbital shaker at room temperature. An equal volume of prewarmed Soybean Trypsin Inhibitor (2.5 mg/ml; Thermo Fisher Scientific) was added to finish trypsinization, and epidermises were rubbed vigorously against bottom of plate using curved forceps to dislodge keratinocytes. Dislodged cells were pooled into a tube on ice in KBM-Gold. Pooled cells were pipetted ~40 times with a 10-ml serological pipette and strained with a 100-μm filter before pelleting cells at 300*g* for 10 min at 4°C. Cells were resuspended in 4.5 ml of KBM-Gold per epidermis, and cell count was obtained using Cellometer ViaStain AOPI Solution (Sigma-Aldrich). The cells were subjected to Percoll (GE LifeSciences; ratio of 10× PBS:Percoll:KBM-Gold media was 1:4:5, where the cells were at a concentration of 1 million per milliliter of final volume) gradient ultracentrifugation using a swinging bucket rotor (42 min at 4°C) to separate the basal and suprabasal fractions. The cells at the top band were suprabasal cells and separated from the pellet at the bottom of the tube, which were basal cells. The basal cell sample was carefully pipetted out, washed twice with 4°C 1× PBS, seeded onto collagen-coated (Collage Type I; Corning) six-well plates at 1 × 10^6^ cells per well, and incubated at 37°C overnight to allow cells to attach to the plate before proceeding with experiments. To estimate mRNA half-lives in skin cells we used basal cells.

Mast cells: Mouse bone marrow–derived mast cells were differentiated from the marrow of tibias and femurs of C57/BL6 mice and cultured for 8 weeks in RPMI 1640 containing 10% FBS, 25 mM Hepes (Gibco), penicillin (100 U/ml), streptomycin (100 μg/ml; Gibco), 2.5 mM L-glutamine, (Gibco), 1 mM sodium pyruvate (Gibco), nonessential amino acids (Gibco), 50 μM 2-mercaptoethanol, IL-3 (20 ng/ml), and stem cell factor (SCF) (20 ng/ml).The purity of mast cells was monitored by assessing the expression of the receptors for SCF (CD117) and for immunoglobulin E (FcεRI) by flow cytometry. By the end of 6 to 8 weeks in culture, more than 98% of the cultured population was FcεRI^+^/CD117^+^ double-positive mast cells.

All animals were housed in the Association for Assessment and Accreditation of Laboratory Animal Care International–accredited animal housing facilities at the National Institutes of Health (NIH). All animal studies were performed according to the NIH guidelines for the use and care of live animals and were approved by the Institutional Animal Care and Use Committee of National Institute of Arthritis and Musculoskeletal and Skin Diseases (NIAMS) and National Institute of Allergy and Infectious Diseases (NIAID) (A021-03-04).

### HK and TF genes

Human HK genes were identified through literature search and selection of genes reported in two independent genome-wide studies (*86*, *87*). A list of human TF genes was obtained from Lambert *et al.* (*88*).

### RNA sequencing

Cells (0.5 M) were harvested and lysed in 100 μl of lysis solution (RNAqueous-Micro Total RNA Isolation Kit from Invitrogen). RNA was isolated and deoxyribonuclease treated according to the manufacturer’s protocol. High RNA quality was confirmed using the Agilent TapeStation system before library preparation. mRNA polyA purification, reverse transcription, and library preparation were performed using the NEBNext Poly(A) mRNA Magnetic Isolation Module and the NEBNext Ultra II Directional RNA Library Prep Kit for Illumina (New England Biolabs). For spike-in control, ERCC RNA Spike-In Mix (Invitrogen) was added before RNA isolation. Single 50 cycles of sequencing data were acquired on NextSeq 2000, NextSeq 550, HiSeq 3000, NovaSeq 6000, or NovaSeq X Plus (Illumina). RNA-seq was performed in three replicates.

### Chromatin immunoprecipitation sequencing

Cells were fixed with 1% formaldehyde (Sigma-Aldrich) for 10 min at 37°C, treated with 1/20 volume of 2.5 M glycine, washed with PBS, snap frozen on dry ice, and stored at −80°C until further processing. After thawing, cells were resuspended in radioimmunoprecipitation assay (RIPA) buffer [10 mM tris (pH 7.6), 1 mM EDTA, 0.1% SDS, 0.1% sodium deoxycholate, and 1% Triton X-100] supplemented with Complete Mini EDTA free proteinase inhibitor (Roche) and sonicated using ultrasonicator (Covaris) in 1-ml AFA tubes (Covaris): duration (seconds): 1200, peak power: 75, duty % factor: 15, cycles per burst: 1000. Sonicated chromatin was precleared with Dynabeads and incubated overnight at 4°C with rotation with following antibodies: RAD21: ab992 (Abcam); MED26: 13641S (Cell Signaling Technology); TATA-binding protein TBP: ab28175 (Abcam); BRD4: ab128874 (Abcam); and RNA Pol II: ab26721 (Abcam). The next day, samples were washed (10 min at 4°C with rotation): 2× with RIPA buffer, 2× with RIPA buffer +0.3 M NaCl, 2× with 1 ml of LiCl buffer (0.25 M LiCl, 0.5% Igepal CA-630, and 0.5% NaDOC, stored at 4°C), 1× with 1 ml of Tris-EDTA (TE) + 0.2% Triton X-100, and 1× with 1 ml of TE. After washes, beads were resuspended with 100 ul of TE, followed by addition of 3 μl of 10% SDS and 5 μl of Proteinase K (20 mg/ml) and incubated at 65°C for 4 hours. After decross-linking, DNA was purified on columns using a ChIP DNA Clean & Concentrator kit (Zymo Research). In the next step, DNA was quantified using a Qubit system (Invitrogen), and library was prepared using Ovation Ultralow System V2 (Tecan) according to the manufacturer’s protocol. Before sequencing high quality was confirmed using the Agilent TapeStation system. Single 50 cycles of sequencing data were acquired on NextSeq 550 or NovaSeq 6000 (Illumina). ChIP-seq experiments were performed in two replicates.

### mRNA half-life estimation

Cells were treated with actinomycin D (5 μg/ml; Sigma-Aldrich) and harvested (0.5 M cells) at time point 0 hours and up to 24 hours: at 1-, 2-, 4-, 8-, 12-, and 24-hour time points (*89*). After each time point, the viability of cells was checked with fluorescence-activated cell sorting or a Nexcelom cell counter after staining with acridine orange/propidium iodide (Nexcelom Bioscience). Some cell types did not survive longer actinomycin D treatment than 12 hours, so the last collected time point was determined on the basis of cell type viability (details in fig. S1A). To determine mRNA half-lives upon perturbations, cells were treated with 500 μM auxin (IAA) (Sigma-Aldrich) for 12 hours to induce the degradation of AID-tagged RAD21 and MED26, or with 500 nM JQ1 (Sigma-Aldrich). Following the 12-hour treatment, actinomycin D (5 μg/ml; Sigma-Aldrich) was added, and cell pellets were collected. Pellets were lysed in 100 μl of the lysis solution (RNAqueous-Micro Total RNA Isolation Kit from Invitrogen), ERCC RNA Spike-In Mix (Invitrogen) was added, RNA was isolated (RNAqueous-Micro Total RNA Isolation Kit from Invitrogen), and RNA-seq was performed using the NEBNext Poly(A) mRNA Magnetic Isolation Module and NEBNext Ultra II Directional RNA Library Prep Kit for Illumina (New England Biolabs). To estimate half-lives genome-wide, we used our custom code that uses RNA-seq data as an input. After calculating the concentration of mRNA transcript of each gene from ERCC spike-in information, we estimate the decay rate constant (*k*_decay_) by fitting the exponential curve on concentration changes upon time points using SSasymp R function. From the decay constant, we calculated the mRNA half-lives using the following equation: ln(2)/*k*_decay_. For ES cells, we used published mRNA half-lives (*42*).

### Single-molecule RNA fluorescence in situ hybridization

Cells were detached by Accutase treatment (Gibco), washed three times with Hanks’ balanced salt solution (Gibco), and fixed in pellet with 4% paraformaldehyde (Electron Microscopy Sciences) in 1× PBS (Gibco) for 10 min. After fixation, cells were washed three times with 1× PBS and spun down onto a coverslip using a cytocentrifuge. Next, cells were permeabilized in 70% ethanol for 1 hour, washed once for 10 min with 1× PBS, washed once for 5 min with 10% formamide (Invitrogen)/2× SSC (Invitrogen), and hybridization with exonic probes (0.5 μl 12.5 μM Cy3- and 0.5 μl 12.5 μM Cy5-labeled) (Stellaris) in 50 μl of hybridization buffer (10% dextran sulfate (Millipore)/10% formamide/2× (SSC) was performed for 4 hours at 37°C. After hybridization, cells were washed twice for 30 min with prewarmed to 37°C 10% formamide/2× SSC followed by wash with 2× SCC for 5 min and 1× PBS for 5 min. At the end, samples were mounted with ProLong Diamond Antifade Mountant with 4,6-diamidino-2-phenylindole dihydrochloride (Invitrogen). Details about used probes can be found in data S8. smRNA-FISH was performed in two replicates.

### Microscopy and smRNA-FISH analysis

smRNA-FISH samples were imaged using a custom microscope: “RAMM” Rapid Automated Modular Microscope (ASI Imaging) equipped with 40×/1.4 numerical aperture oil immersion objective (Zeiss) [details in the study of Patange *et al.* (*14*)]. Z-stacks with a 0.5-μm step size were acquired to capture whole cells. The stacks of images were converted into a maximum intensity projection. Next, segmentation of cells and nuclei was done using CellProfiler software (*90*), and spots were counted using Localize and FishAuxiliary software (LarsonLab GitHub).

### Single-cell RNA sequencing

Cells were washed twice with 1× PBS containing 0.04% weight/volume bovine serum albumin, resuspended in the same buffer, and encapsulated into droplets using 10x Genomics system. Libraries were prepared using Chromium Single Cell 3′ Reagent Kits (10x Genomics) according to the manufacturer’s protocol. With double 10-bp index cycles, 28 forward and 90 reverse cycles were run on a NovaSeq 6000 machine (Illumina) or HiSeq 3000 (Illumina).

### Quality control of scRNA-seq data (first quality control step)

We used a 10x Genomics CellRanger pipeline (v3.0.2) and bcl2fastq (v2.20) to generate and align fastq files to the human (hg19) and mouse (mm10) genomes. The filtered gene-barcode matrix data from the Cell Ranger served as an input for Seurat R package (*91*). To eliminate low-quality cells, empty droplets, doublets/multiplets or dying cells from the scRNA-seq data, we assessed the distributions of (i) number of unique genes detected in each cells (feature count), (ii) total number of molecules detected (RNA count) in each cell, and (iii) the percentage of reads that map to the mitochondrial genome. We applied stringent filtering to exclude cells located in the extreme tails of each distribution, retaining cells primarily within approximately 90% of the distribution centered around the middle. These filtering parameters were adjusted on the basis of the cell type and sequencing depth. Furthermore, we excluded unhealthy cells by analyzing only those with mitochondrial counts below 5 to 7.5%.

### Model used to infer bursting kinetics

We fit the classic two-state stochastic telegraph model to the scRNA-seq data for each gene. The model consists of transitions between an OFF state and ON state, where mRNA are created and emitted in the ON state and then disappear. Each transition is entirely stochastic with a transition rate parameter. There are four total parameters, which we call the ON rate, *k*_on_ (OFF to ON transition), OFF rate, *k*_off_ (ON to OFF transition), eject rate, *k*_eject_ (mRNA creation rate), and decay rate, *k*_decay_ (mRNA disappearance rate). From these rates, we also compute the “burst size,” *k*_eject_/*k*_off,_ which corresponds to the mean number of mRNA produced while in the ON state. We note that this burst size does not necessarily correspond to what is measured in live-cell recordings because it does not account for the stochastic nature of the pre-RNA kinetics (*7*, *39*). Perhaps confusingly, in the two-state telegraph model, the mean time the gene is OFF is given by 1/*k*_on_ (because the OFF duration is the time it takes to turn on), while the mean ON duration is 1/*k*_off_. The OFF duration is also called the burst frequency. As a control, we also fit one-state (no OFF states) and three-state models (two OFF states).

The probability distribution of the system is governed by the chemical master equation (CME). We assume that the scRNA count histograms are in steady state that can be compared to the scRNA probability distribution from solving the CME. This is difficult to do in general because there are a countably infinite number of mRNA states. We make this problem tractable by noting that the probability distribution is concentrated at low values and truncate the infinite dimensional system. This reduces the problem to finding the null space of a finite-sized transition matrix, which can be solved quickly using QR decomposition. The advantage of solving the CME directly rather than using the Beta-Poisson representation is that the same algorithm can be used for any discrete stochastic Markov process. For example, the same algorithm was used to solve generalized multistate telegraph models as well as models that include elongation and splicing steps (*7*, *39*).

We estimate the posterior distributions for the rate parameters using a Bayesian Metropolis-Hastings MCMC algorithm. The estimation is confounded by three important issues. The first is that the steady-state mRNA distribution has no timescale and is thus only defined by three of the four rate parameters of the telegraph model. In the past, this has often been handled by fixing the decay rate and thus estimating the other three rates in terms of the decay rate. We resolve this issue by using independently measured half-lives of each gene as a prior of the scRNA decay rate. The second issue is that only a small fraction of the mRNA (which we call the yield) is captured by the scRNA measurement. We estimate the yield by comparing scRNA to FISH for a number of genes. We find that the yield can vary between genes and, and when averaged over, our measured genes was approximately 5%. It can be proven for any generalized telegraph model with an arbitrary number of states that if the experimental collection of mRNA is completely random and governed by a binomial or Poisson distribution, then the yield only affects the eject rate (see below for proof). The third issue is that genes usually have more than one allele transcribing the gene simultaneously. In the computations, we assumed the alleles are uncorrelated and then computed the multiallele probability density by convolving the single allele density. We used Hi-C data to determine the number of alleles in immortalized cell lines: HCT-116 (*52*) and CH12 cells (*58*). To do this, we used HiNT package (*92*), a tool designed to predict copy numbers using Hi-C contact matrix data. In all other cases, we assumed the presence of two alleles.

We assume that each mRNA count value per cell is independent, and thus, the likelihood function is the product of the predicted probability at the values of all the data points. The log of the likelihood function is thus identical to the cross entropy between the predicted distribution and the scRNA count histogram. All rate units were taken to be in minutes. We use broad priors for the three parameters (and fix the decay rate at the measured value) using values from previous fits to live-cell data. Specifically, the priors are log normal distributions with means 0.01 for the ON rate, 0.1 for the OFF rate, and 0.05 (equivalent to 1 times the yield prior) for the eject rate. We used a coefficient of variation of 10 as the prior variances.

The MCMC was run as two to eight chains (usually four) for up to 10 million samples. We monitored convergence by using $\hat{r}$ (*93*). After an initial run, genes that had $\hat{r}$ far from one were rerun until they neared one. All computations were performed in a Julia software package we created called StochasticGene.jl, which can be installed directly from Julia. The code is open source and available with documentation at (https://github.com/nih-niddk-mbs/StochasticGene.jl). The package is frequently updated; the fits in the paper used version 0.7.8. The package can fit a wide range of stochastic models to both mRNA count data (FISH or scRNA) along with ON and OFF distributions and spot intensity time series from live-cell recordings. We used the median of the posteriors for values in the graphs and the MAD or 95% posterior credible intervals for the uncertainty. The uncertainty for the burst size was computed by propagating errors from the joint posterior of the OFF and eject rates and accounts for the cross-correlations. We also computed maximum likelihood estimates as a comparison to the median values.

### Proof that random loss of scRNA results in rescaling of eject rate

Proposition: Suppose the steady-state probability distribution for mRNA obeys the Poisson-Beta distribution *P*(*m*) but the probability of observing a molecule is *p* < 1 and obeys a binomial distribution, i.e., *m* ~ Bin(*m*’,*p*), *m*’ ~ *P*(*m*’). Then the eject rate ν will be rescaled by the loss probability *p*, while the other parameters remain unaffected.

Proof:

The observed probability mass function *P*(*m*) will be

$$P\left(m\right)=\sum_{n=0}^{\infty }\mathrm{Bin}\left(m|n,p\right)\;P\left(n\right)\;\left(*\right)$$

where Bin is the binomial distribution

$$\mathrm{Bin}\left(m|n,p\right)=\sum_{n=0}^{\infty }\frac{n!}{\left(n-m\right)!m!}p^{m}{\left(1-p\right)}^{n-m}$$

and the mRNA distribution of the two-state telegraph model, *P*(*n*), obeys a Poisson-Beta distribution

$$P\left(n\right)=B\left(\alpha ,\beta \right)\int_{0}^{1}\lambda^{\alpha -1}{\left(1-\lambda \right)}^{\beta -1}\frac{{\left(\nu \lambda \right)}^{n}e^{-\nu \lambda }}{n!}d\lambda$$

where α and β are the ON and OFF transition rates, and ν is the eject rate.

Inserting both distributions into (*), performing the sum, and rearranging give

$$P\left(n\right)=B\left(\alpha ,\beta \right)\int_{0}^{1}\lambda^{\alpha -1}{\left(1-\lambda \right)}^{\beta -1}\frac{{\left(p\nu \lambda \right)}^{m}e^{-p\nu \lambda }}{m!}d\lambda$$

Thus, the eject rate is rescaled by the technical loss ν → *p*ν.

### Quality control of inferred rates (second quality control step)

Only genes that passed quality control were analyzed. The filtering was performed on the basis of the following criteria: (*k*_on_ MAD/*k*_on_ Median) < 0.75, *k*_off_ MAD/*k*_off_ Median) < 0.75, (*k*_eject_ MAD/*k*_eject_ Median) < 0.75, (Burst Size MAD/Burst Size Median) < 0.75, Expression >0.01.

### Cell cycle analysis

On the basis of 10x scRNA-seq expression of cell cycle markers, we sorted HCT-116 cells into G_1_, S, and G_2_-M stages. Moreover, to avoid bias caused by not equal number of cells, we matched the number of cells in all stages. Next, we fitted the model and correlated all rates inferred from cells in different cell cycle stages.

### Analysis of bursting across cell types

To calculate normalized burst size and expression, we used the following formula for normalization

$$\frac{\text{Expression or burst size}}{\text{Reads}\;\text{per}\;\text{cell}\;}\times \text{Average reads}\;\text{per}\;\text{cell across cell types}$$

The normalization was applied to the analysis in Fig. 3 (D to F) and fig. S5 to facilitate the comparison of rates across different cell types.

### Differentially expressed gene analysis of mRNA-seq data

Reads were aligned to the mouse genome (NCBI37/mm9) or the human genome (hg19) with GSNAP without detecting splice junctions de novo (--novelsplicing = 0) (*94*). Existing splice junctions from RefSeq annotation were taken into account (−-use-splicing=/path/to/mm9.splices.iit). Output files were filtered to remove unaligned reads and any alignments with a mapping quality less than 20. Reads were mapped to RefSeq genes with htseq-count -m intersection-nonempty and basemean, FC and adjusted *P* values for the differentially expressed gene analysis were calculated using the R package DESeq2 (*95*). For mRNA-seq data, genes with more than 100 base mean, greater than 1.5 FC, and less than 0.01 adjusted *P* values were selected as the significantly differentially expressed genes. Genes down-regulated upon *Myc* deletion were identified using scRNA-seq with a 1.25-FC threshold and adjusted *P* < 0.01. Down-regulated genes following RAD21, MED26 removal, and JQ1 treatment were identified on the basis of bulk mRNA-seq data with a 1.5-fold threshold and adjusted *P* < 0.01. All other genes, except those significantly down-regulated, were classified as “other.” These categories were then applied to the analysis of bursting parameters.

### ChIP-seq analysis

Sequence reads were aligned to the mouse genome (NCBI37/ mm9) or the human genome (hg19) using bowtie with flags -S -m 1 -a --best --strata -n 2, and aligned reads were selected with samtools view -S -b -F4 and sorted (*96*, *97*). Using Picard (http://broadinstitute.github.io/picard), we removed duplication, and then we extended the reads into the estimated fragment sizes by MaSC. MACS2 was used for peak calling with corresponding input files and 0.001 *P* value cutoff for each ChIP-seq (*98*). For comparison between conditions, we merged the peaks from each condition by using bedtools merge function (*99*). ChIP-seq signals were counted on the merged peak regions which are categorized into promoter, enhancer, SE, and others if necessary. Promoter regions are defined by the transcription start site (TSS) +/− 1 kb, and enhancer regions are defined by the accessibility and H3K27ac ChIP-seq peaks. SE regions are defined by the ROSE program (*53*, *100*), which is using H3K27ac ChIP-seq data. For the RNAP2 signal composite analysis around genes, we created 100-nt windows in the upstream and downstream 5-kb regions of genes and 100 bins in the gene-body region with various sizes according to the gene length for down-regulated genes or other genes separated by more than 5 kb from neighboring genes. Using “Bedtools coverage” function with counts option (*99*), we calculated the signals within those windows. After computing RPKM values at each position, we obtained mean values after trimming 10% of outliers.

### Precision nuclear run-on sequencing

HCT-116 cells were treated in the same way as for scRNA-seq (Fig. 4A) and ChIP-seq (Fig. 5 and fig. S13) experiments. Briefly, cells were serum starved for 14 hours, followed by incubation with FBS-free medium for 6 hours in the presence of DMSO (control), 500 μM auxin (Sigma-Aldrich) for degradation of the AID-tagged MED26, or with 500 nM JQ1 (Sigma-Aldrich). PRO-seq library construction and data analysis was performed by the Nascent Transcriptomics Core at Harvard Medical School, Boston, MA. Thawed aliquots of permeabilized cells, previously stored at −80°C, were gently pipetted on ice until fully resuspended. Subsequently, permeabilized cells were counted using a Luna FX7 (Logos Biosystems) instrument. For each sample, 1 million permeabilized cells were used for nuclear run-on, with an additional 50,000 permeabilized *Drosophila* S2 cells added for normalization. Nuclear run-on assays, library preparation and data analysis followed the protocol outlined by Mimoso and Goldman (*101*). Pooled libraries were then sequenced using a NextSeq 2000 (Illumina). For PRO-seq composite analysis, we used the same strategy as that used in the RNAP2 ChIP-seq composite analysis. The difference lies in our utilization of strand-sensitive windows with a strand-sensitive bedgraph to obtain sense-stranded signals for PRO-seq. To calculate the signals from the bedgraph, we used the “bedtools intersect” function (*99*). To compute the pausing index, we adhered to the definition and description outlined in the NRSA package (*68*). Promoter regions were defined up to +/− 500-bp regions from the TSS, and gene body regions were defined from 1 kb downstream of the TSS to the transcription end site. The pausing index represents the ratio of the maximum promoter signal over the total gene body signals. Maximum promoter signals were selected from among 50-bp windows in the promoter region with a sliding 5-nt step. PRO-seq experiments were performed in two replicates.

### Gene network analysis

After quality control of scRNA-seq data (first quality control step), we normalized the data to minimize error due to technical variation and to allow the comparisons of samples with differing sequencing depth. We followed a similar simple normalization protocol that was shown to minimize bias when performing covariance analysis on scRNA-seq data (*102*). To minimize variability per cell, we converted the number of reads of a gene per cell to the fraction of reads per cell by dividing by the total number of reads per cell. Second, to convert back to counts while adjusting for the differences in sequencing depth between the samples, we multiplied by the average number of reads per cell in the sample with the lowest sequencing depth; the value was ~30,000 (determined by the RAD21 degron condition) and equates to reads per 30,000. To maintain the discretization of the data, we then rounded any fractional values. For further covariance analysis, we only used pairs of genes where both were detected in at least 10% of the cells. Covariances were defined as the Spearman’s rank correlation coefficient and calculated with the SciPy Python package.

### Figures

The schematic cartoons of Figs. 1A, 3A, and 4A and fig. S2 were created with BioRender.com.

### Statistical analysis

Analysis was done using Kolmogorov-Smirnov and Wilcoxon test in R; details can be found in the figure legends.
