# Supplementary material for: 3D chromatin architecture, BRD4, and Mediator have distinct roles in regulating genome-wide transcriptional bursting and gene network
Source: Sci Adv. 2024 Aug 9;10(32):eadl4893. doi: 10.1126/sciadv.adl4893 (PMC11313860; doi:10.1126/sciadv.adl4893)
Supplement: Supplementary file 1 — Figs. S1 to S14 Legends for data S1 to S8 [file sciadv.adl4893_sm.pdf]

Science Advances
AAAS

Supplementary Materials for

3D chromatin architecture, BRD4, and Mediator have distinct roles in regulating genome-wide transcriptional bursting and gene network

Pawel Trzaskoma *et al.*

Corresponding author: Pawel Trzaskoma, pawel.trzaskoma@nih.gov; Carson C. Chow, carsonc@niddk.nih.gov; John J. O'Shea, osheaj@arb.niams.nih.gov

Sci. Adv. **10**, eadl4893 (2024)
DOI: 10.1126/sciadv.adl4893

The PDF file includes:

Figs. S1 to S14
Legends for data S1 to S8

Other Supplementary Material for this manuscript includes the following:

Data S1 to S8

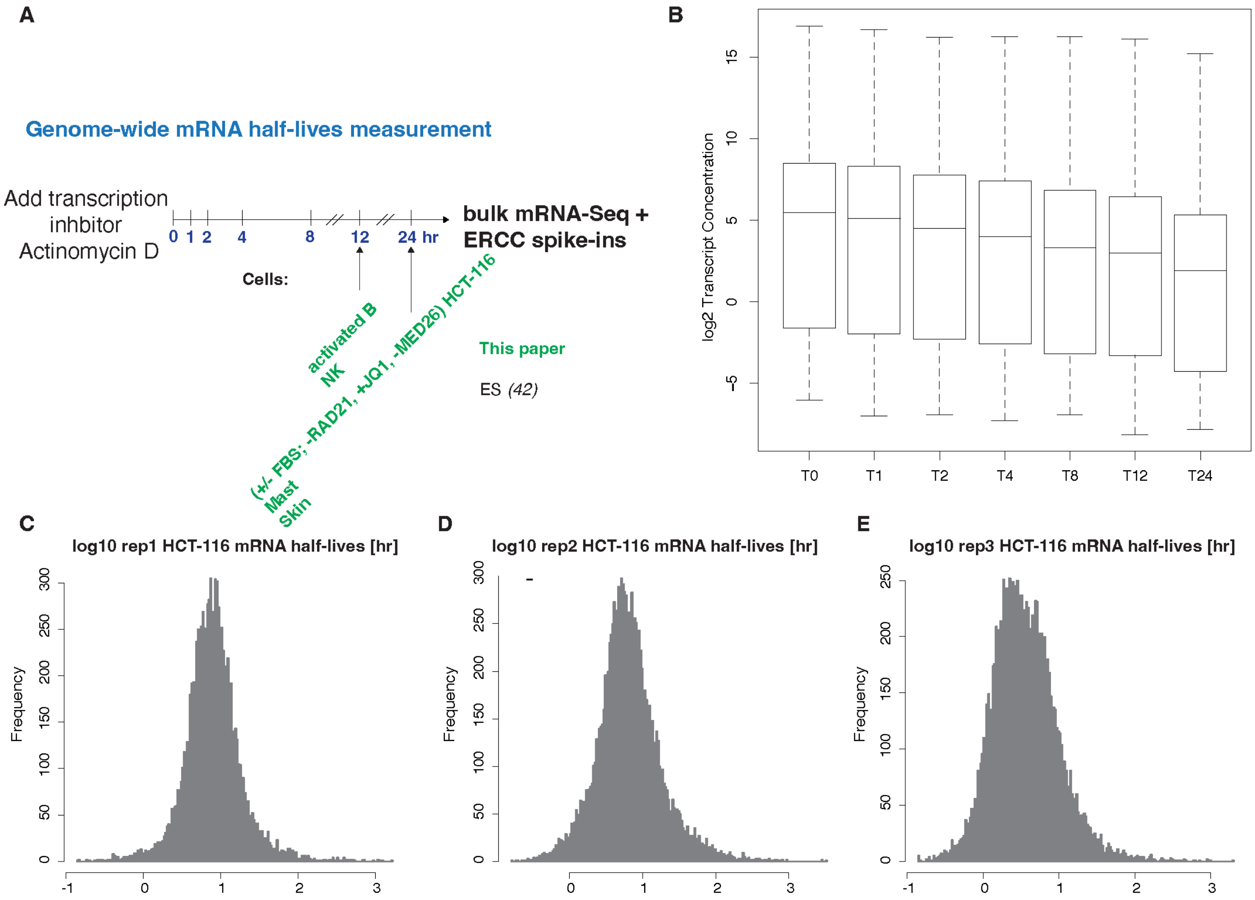

Fig. S1.

Transcription-wide measurements of mRNA half-lives. (A) Experiments performed to measure half-lives. **(B)** Transcript concentration based on bulk mRNA-Seq after 0 and 1, 2, 4, 8, 12 and 24 hours of treatment with 5 µg/mL actinomycin D. **(C-E)** mRNA half-lives [hr] in HCT-116 cells.

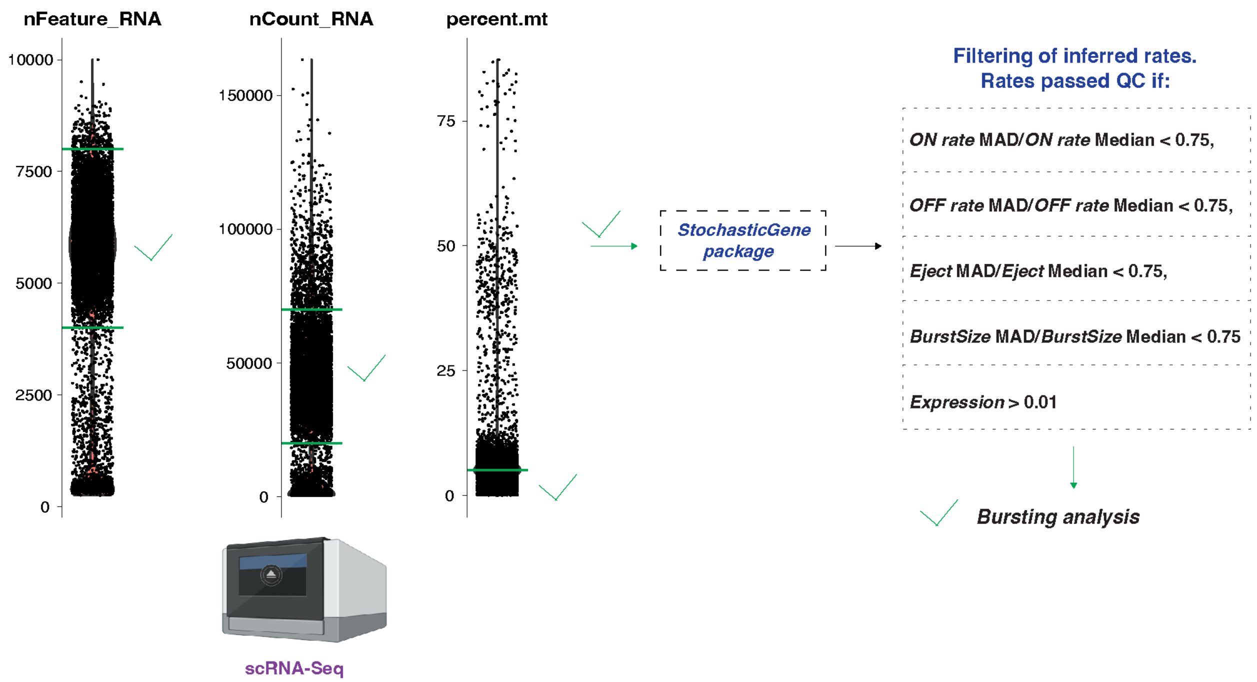

Fig. S2.

Quality control of scRNA-Seq data and inferred rates. Before fitting the model, scRNA-Seq data is filtered based on number of detected genes (nFeature_RNA), total number of molecules identified within a cell (nCount_RNA) and percentage of reads mapped to the mitochondrial genome (percent.mt). The filtered data is used as an input for the model. After the fitting, additional filtering is performed based on a median absolute deviation (MAD) and expression.

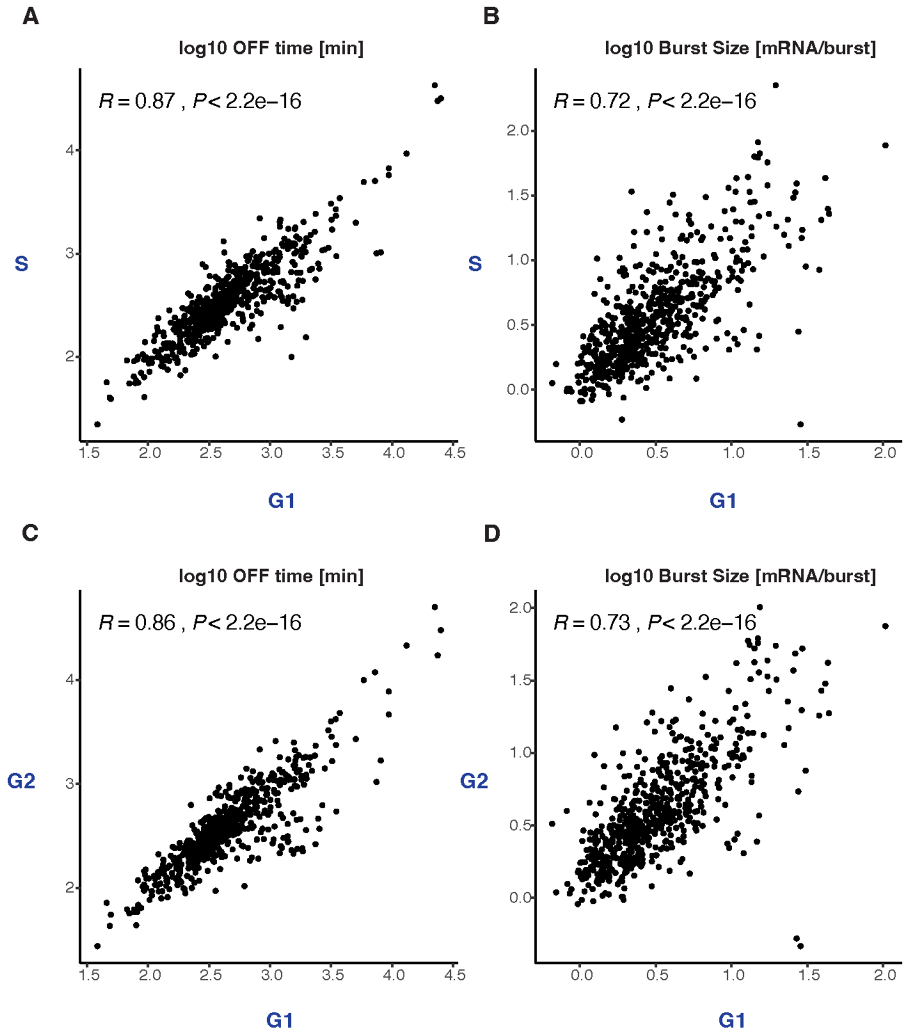

Fig. S3.

Transcription bursting inferred from cells in G1, S and G2 cell cycle stages. (A) OFF duration S vs. G1. **(B)** burst size S vs. G1. **(C)** OFF duration G2 vs. G1. **(D)** burst size G2 vs. G1. All rates inferred in HCT-116 cells from the same number of G1, S and G2 cells. *Pearson R, P values as shown, n = 607.*

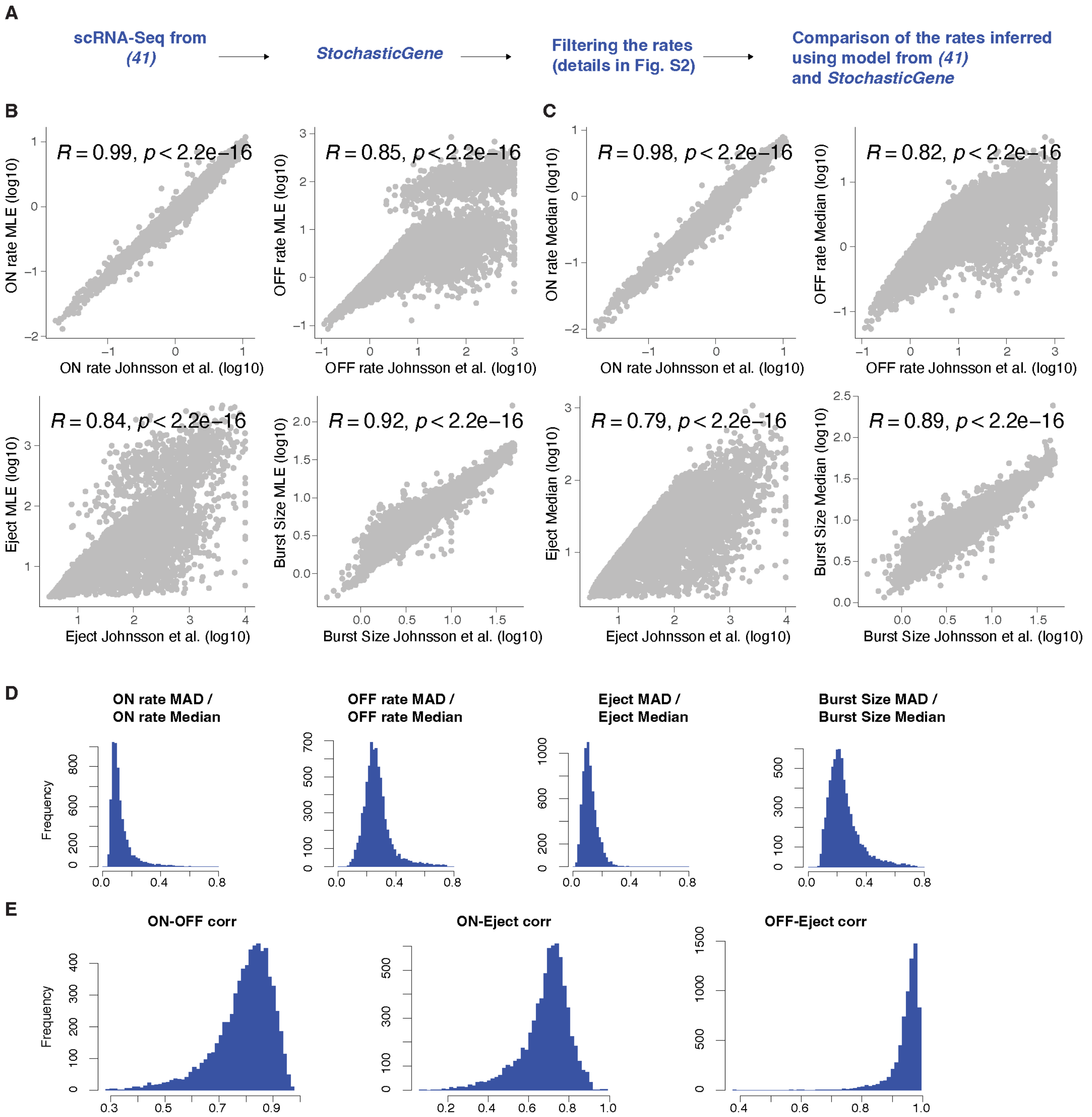

Fig. S4.

Validation of StochasticGene. (A) The StochasticGene pipeline was employed to model scRNA-Seq data obtained from Johnsson et al. (*41*). After quality control and gene filtering, we computed the Spearman's correlation coefficients (*R*) between the remaining rate estimates. We set the decay rates to one for all genes as was done in (41). **(B)** Maximum Likelihood Estimation (MLE), and **(C)** Median posterior rates inferred with 2-state telegraph model (StochasticGene) were compared to rates inferred with the estimates from Johnsson et al. (*41*). **(D)** Histograms displaying median posterior rates / median absolute deviation (MAD) values for these rates, based on StochasticGene and scRNA-Seq from Johnsson et al. (*41*). The posteriors are wider for k_{off} and k_{eject} than k_{on} , consistent with the higher correlation for k_{on}.**(E)** Histograms illustrating the correlation between rates, based on StochasticGene and scRNA-Seq from Johnsson et al. (*41*). The high correlation

between k_{off} and k_{eject} indicates that there is a trade-off between the two variables and further explains why the correlation for burst size (which is the quotient of the two rates) was higher than for k_{off} and k_{eject} alone.

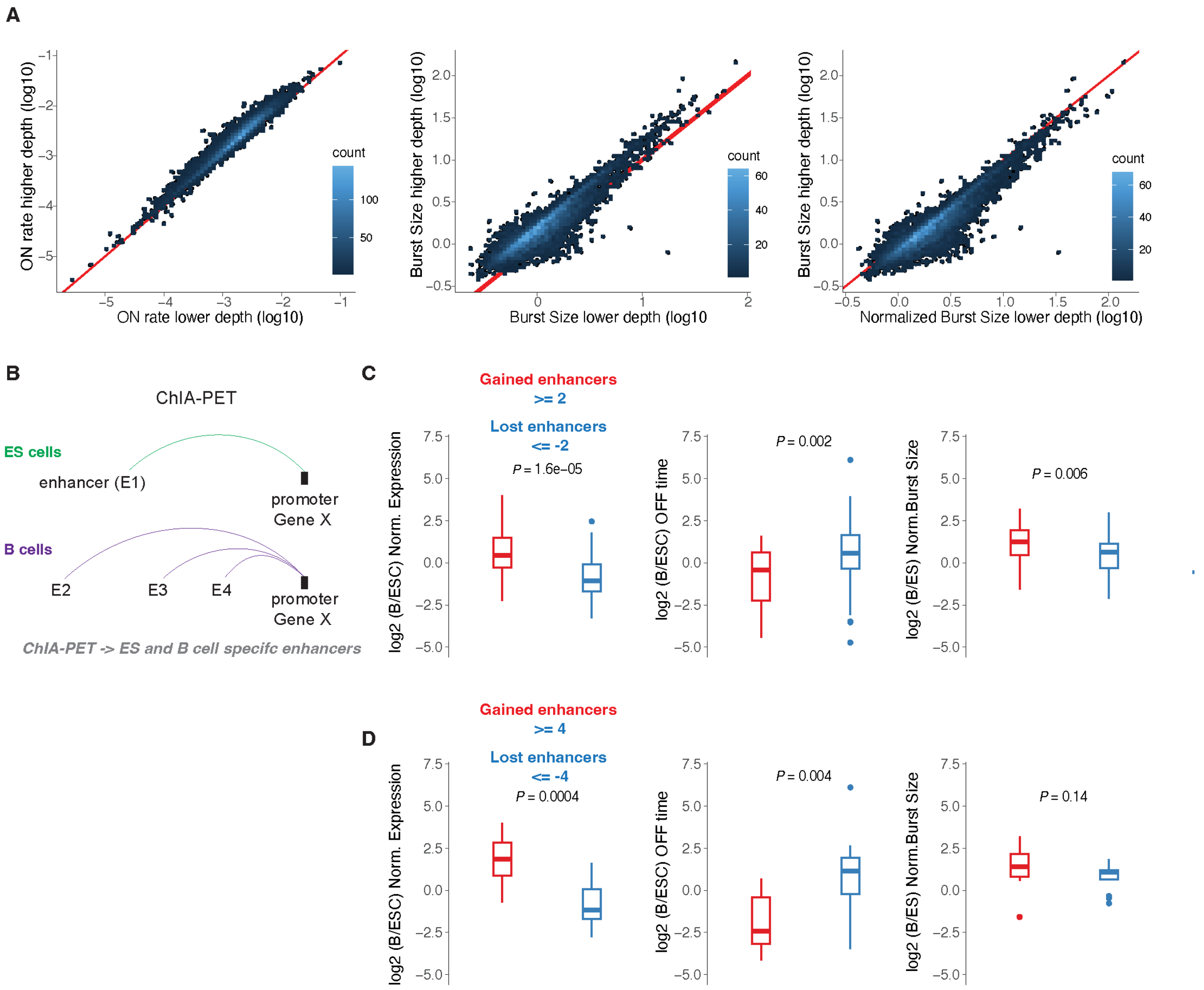

Fig. S5.

(related to Fig. 3). Expression gained during cell development (ES -> B cells) driven by ChIA-PET revealed enhancers, is dictated by burst frequency, and to some extent by burst size. **(A)** The same sample was sequenced twice to assess the impact of higher sequencing depth on k_{on} and burst size. The telegraph model was fitted to the sample after the initial round of sequencing (lower depth: ~42,760 reads per cell) and again after the second round of sequencing (higher depth: ~79,328 reads per cell). The plots illustrate parameters inferred from samples with higher versus lower sequencing depth for k_{on} (left panel), burst size (middle panel), and burst size versus normalized burst size (right panel). Normalization by sequencing depth mitigated the shift induced by varying sequencing depths. **(B)** ChIA-PET revealed cell-type specific promoter-enhancer contacts in ES and B cells. **(C-D)** LFC (B/ES) of normalized expression, OFF duration, and normalized burst size of genes with entirely changed cell-type specific enhancer landscape during B cell development. **(C)** gained (associated with ≥ 2 cell-specific enhancers) and lost (≤ -2 cell-specific enhancers). **(D)** gained (associated with ≥ 4 cell-specific enhancers) and lost (≤ -4 cell-specific enhancers); based on ChIA-PET (*46*), *two-sided unpaired Wilcoxon test, P values as shown,* B*: n = 29 gained and 62 lost,* C*: n =10 gained and 15 lost.*

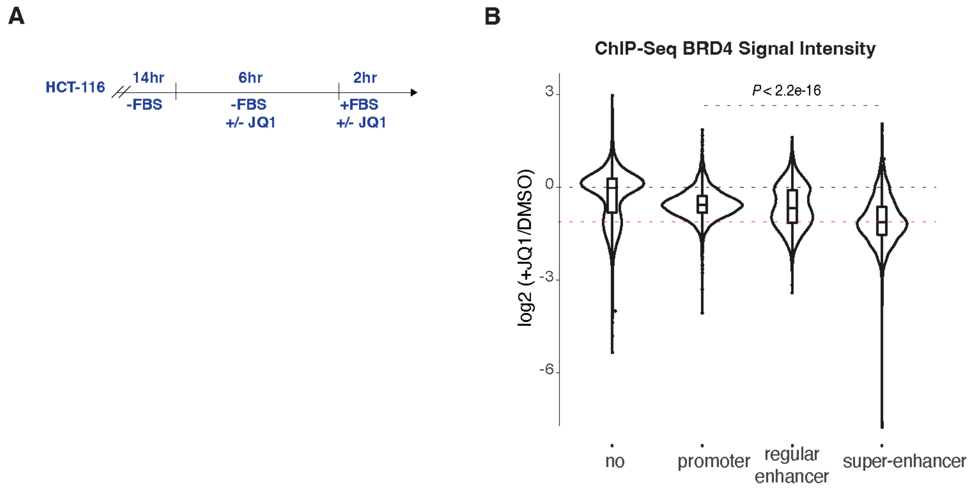

Fig. S6.

(related to Fig. 4). JQ1 treatment affects binding of BRD4 mostly at super-enhancers. (A) Experimental design of JQ1 treatment (500nM) in HCT-116 cells. **(B)** log2 (+JQ1/DMSO) ChIP-Seq BRD4 signal intensity at promoter (P, *n = 12,765 peaks*), regular enhancer (RE, *n = 9,506 peaks*), super-enhancer (SE, *n = 2,262 peaks*) and no (*n = 15,549 peaks*) – other than P, RE, SE, *two-sided unpaired Wilcoxon test, P values as shown.*

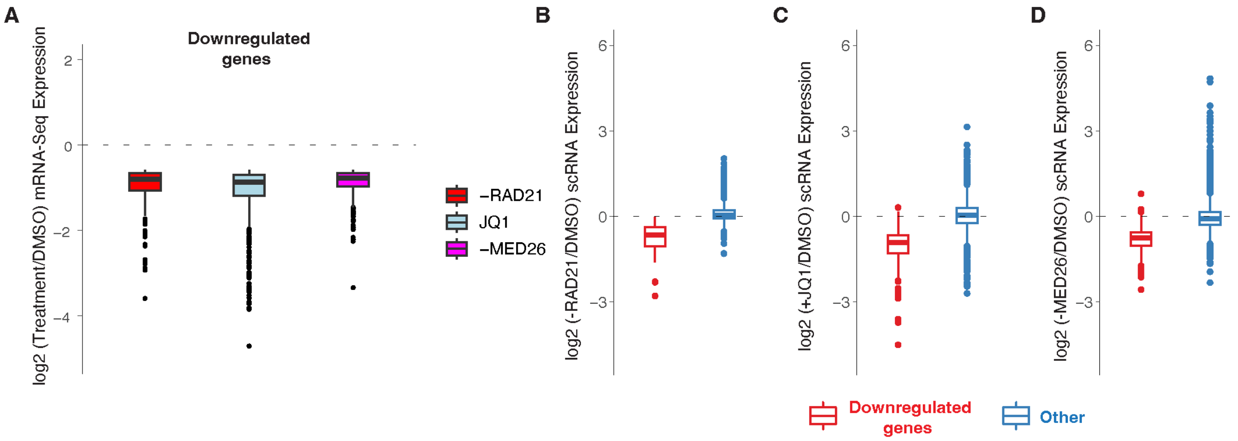

Fig. S7.

(related to Fig. 4). Cohesin, BRD4 and MED26 perturbations have comparable effects on RNA levels. (A) LFC (Treatment/DMSO) Expression based on bulk mRNA-Seq upon RAD21 loss (in red, *n = 416*), JQ1 treatment (in blue, *n = 1,192*), and MED26 loss (in magenta, *n = 1,149*) for significantly downregulated genes. **(B)** LFC (-RAD21/DMSO) Expression based on scRNA-Seq upon RAD21 loss. **(C)** LFC (+JQ1/DMSO) Expression based on scRNA-Seq upon JQ1 treatment. **(D)** LFC (-MED26/DMSO) Expression based on scRNA-Seq upon MED26 loss. **(B-D)** same genes as in Fig. 4B-C; *in red: significantly downregulated genes, in blue: other genes, based on bulk mRNA-Seq.*

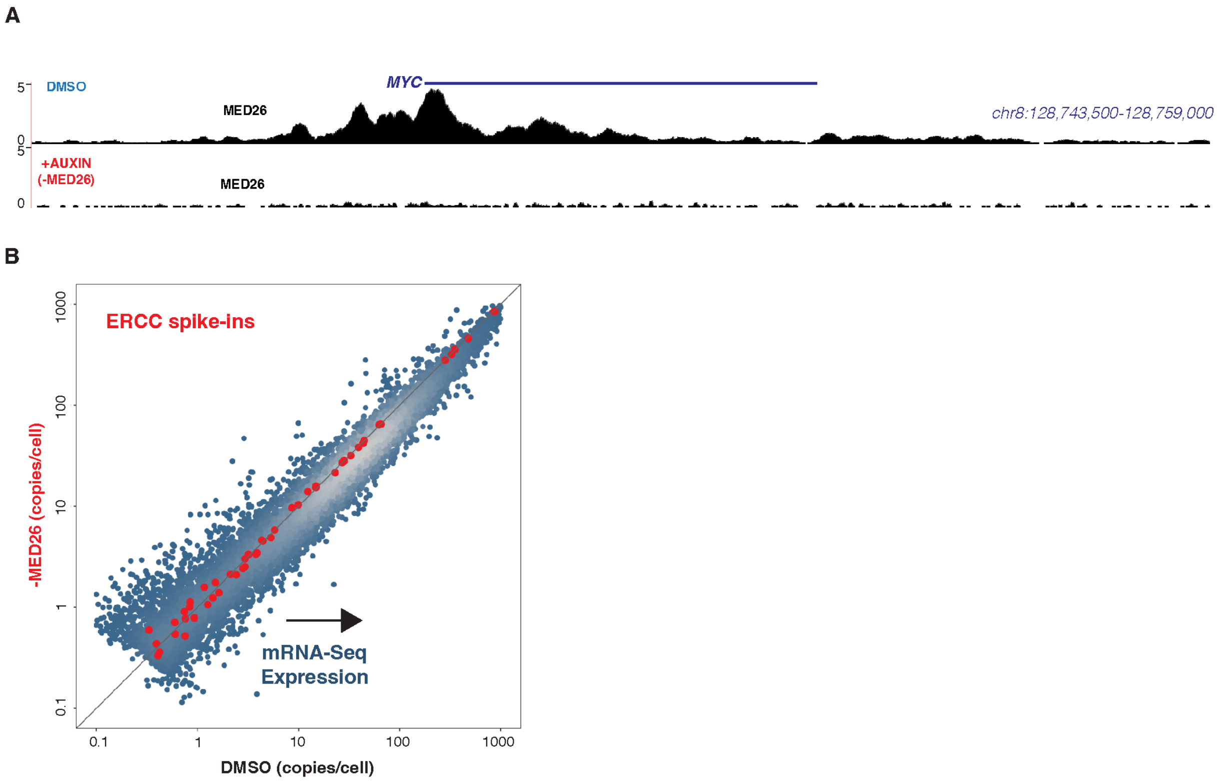

Fig. S8.

(related to Fig. 4). ChIP-Seq validation of MED26 loss in HCT-116 degron cells. (A) Screenshot of genome browser presenting MED26 ChIP-Seq binding tracks in control (DMSO) and MED26 depleted cells +AUXIN (-MED26), *chr8:128,743,500-128,759,000, scale 0-5.* **(B)** Scatter plot presenting results of bulk mRNA-Seq (copies/cell): MED26 depleted cells vs. control cells (DMSO). Red dots indicate ERCC spike-ins control.

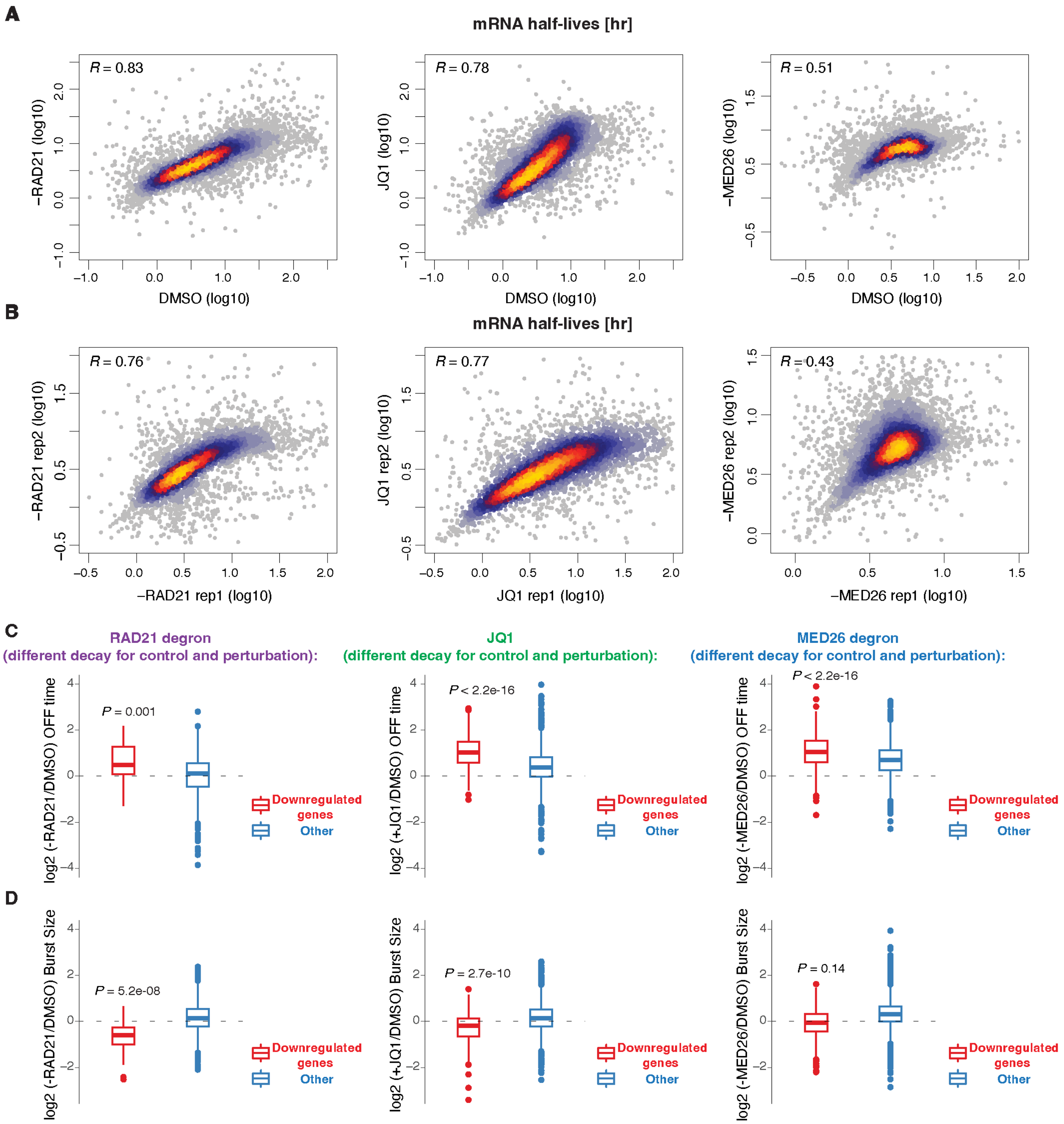

Fig. S9.

(related to Fig. 4). Impact of decay rates on estimated bursting parameters upon cohesin, BRD4 and MED26 perturbations. (A) Correlation of mRNA half-lives upon perturbations of RAD21 (left panel), BRD4 (middle panel; JQ1 treatment), and MED26 (right panel) versus mRNA half-lives measured in control samples (DMSO) in HCT-116 cells. *Two biological replicates were merged; Spearman R; n = 9,399 genes (RAD21), 8,082 genes (JQ1), 11,227 genes (MED26).* **(B)** Correlation of mRNA half-lives between two replicates upon perturbations of RAD21 (left panel), BRD4 (middle panel; JQ1 treatment), and MED26 (right panel). *Spearman R; n = 6,411 genes (RAD21), 7,068 genes (JQ1), 9,374 genes (MED26).* **(C-D)** A two-state telegraph model was fitted to the scRNA-Seq data to infer parameters of bursting in control and treated cells using different decay rates for both conditions. To assess the impact of mRNA half-life differences, the same

genes (with resolved decay rates for both control and perturbation) are shown as in Fig. 4C-D. **(C)** LFC of OFF time duration of significantly downregulated genes (in red) upon RAD21 loss *(n = 49)*, JQ1 treatment *(n = 255)*, MED26 loss *(n = 549)*, and other genes (in blue). **(D)** LFC of burst size of significantly downregulated and other genes. *Two-sided paired Wilcoxon test, P values as shown.*

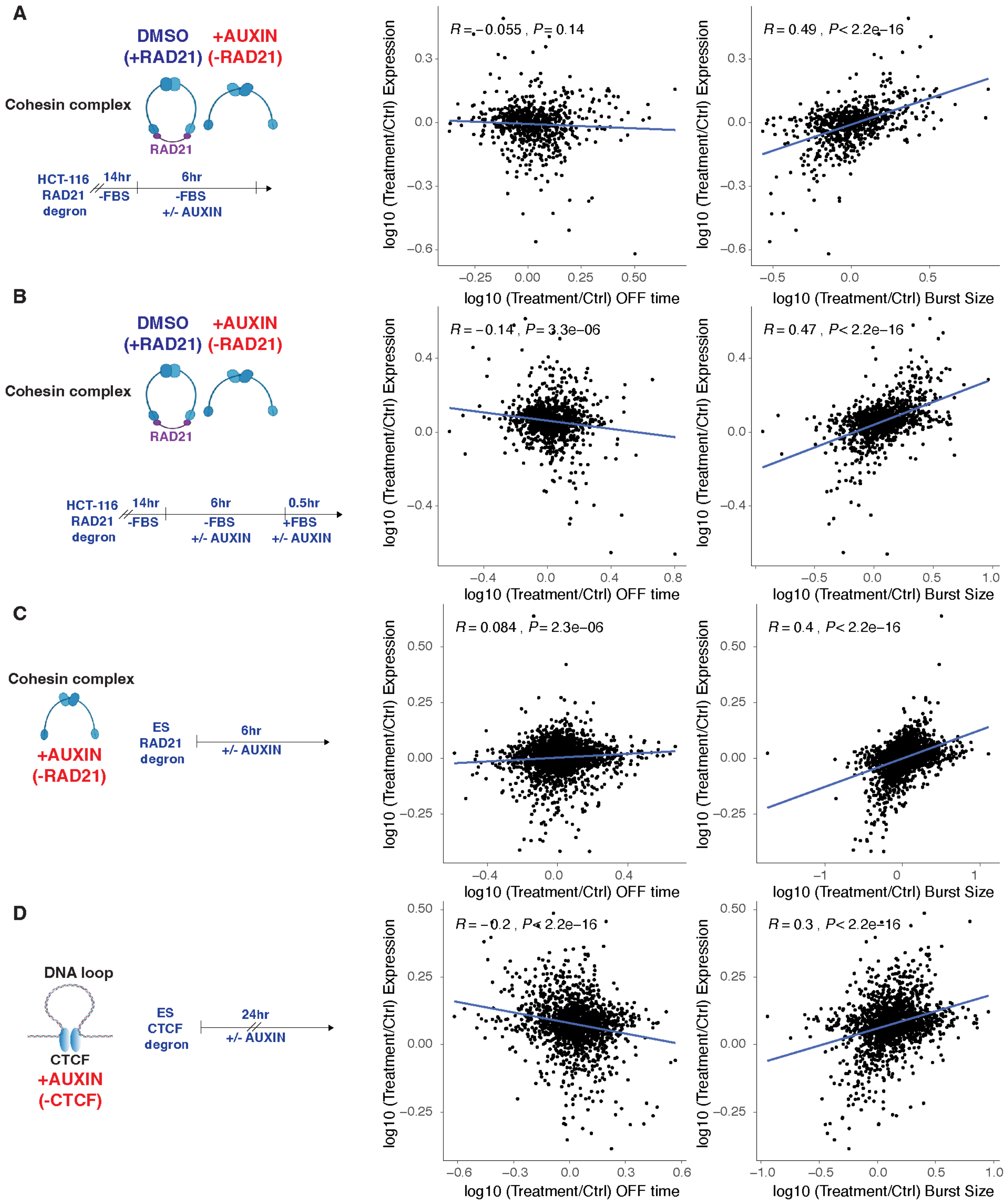

Fig. S10.

(related to Fig. 4). Impact of 3D chromatin architecture by cohesin and CTCF depletion on bursting kinetics. Each panel (A-D) presents experimental design for perturbation (left side), followed by LFC expression vs. LFC OFF duration (middle panel) and LFC of expression vs. LFC of burst size (right panel), *Pearson R, P values as shown.* **(A)** Cohesin removal in serum starved

HCT-116 RAD21 degron cells *(n = 729)*. **(B)** Cohesin removal in serum starved and activated (0.5h) HCT-116 RAD21 degron cells *(n = 1,154)*. **(C)** Cohesin removal in mouse ES RAD21 degron cells *(n = 3,162)*. **(D)** CTCF removal in mouse ES CTCF degron cells *(n = 1,632)*. LFC means log10 change (Treatment/Control), *blue lines represent regression fit.*

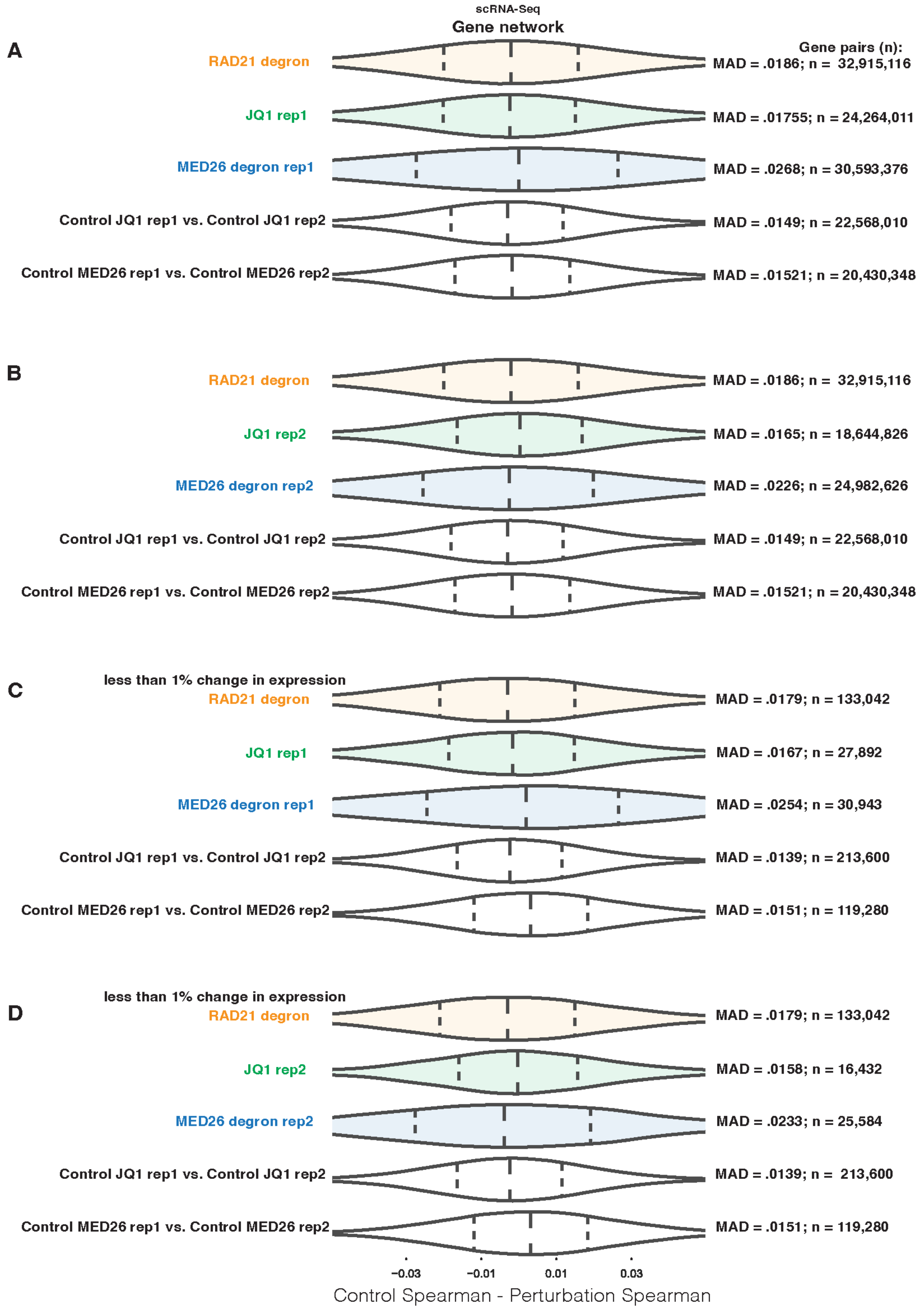

Fig. S11.

(related to Fig. 5). Impact of cohesin, JQ1 treatment and MED26 on gene network. (A-B) Impact of tested perturbations on gene-gene correlations across single cells based on scRNA-Seq;

MAD: median absolute deviation; replicate 1 and 2; *n represents number of gene pairs*. **(C-D)** like A-B but analyzed only genes with less than 1% change in expression.

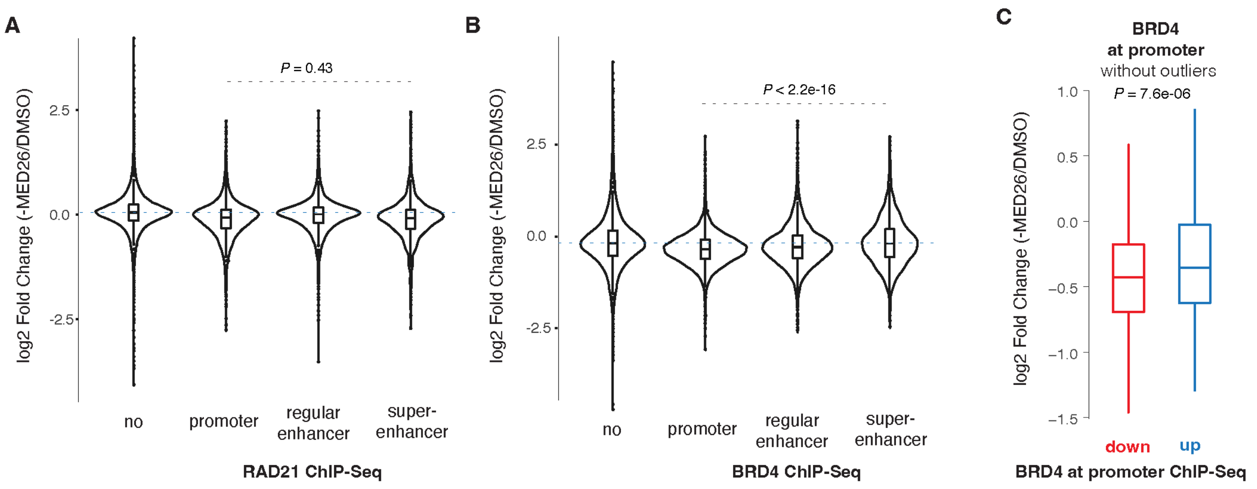

Fig. S12.

(related to Fig. 5). Impact of MED26 loss on RAD21 and BRD4 binding. (A) LFC of RAD21 ChIP-Seq signal intensity at promoter (P, *n = 8,784 peaks*), regular enhancer (RE, *n = 9,872 peaks*), super-enhancer (SE, *n = 1,759 peaks*) and no (*n = 41,428 peaks)* – other than P, RE, SE, *two-sided unpaired Wilcoxon test, P values as shown.* **(B)** LFC of BRD4 ChIP-Seq signal intensity at P (*n = 12,632 peaks)*, RE (*n = 9,352 peaks)*, SE *(n = 1,882 peaks)* and no (*n = 15,460 peaks*) – other than P, RE, SE, *two-sided unpaired Wilcoxon test, P value as shown.* **(C)** LFC of BRD4 at promoter of downregulated (down) and upregulates (up) genes, *two-sided unpaired Wilcoxon test, P values as shown.*

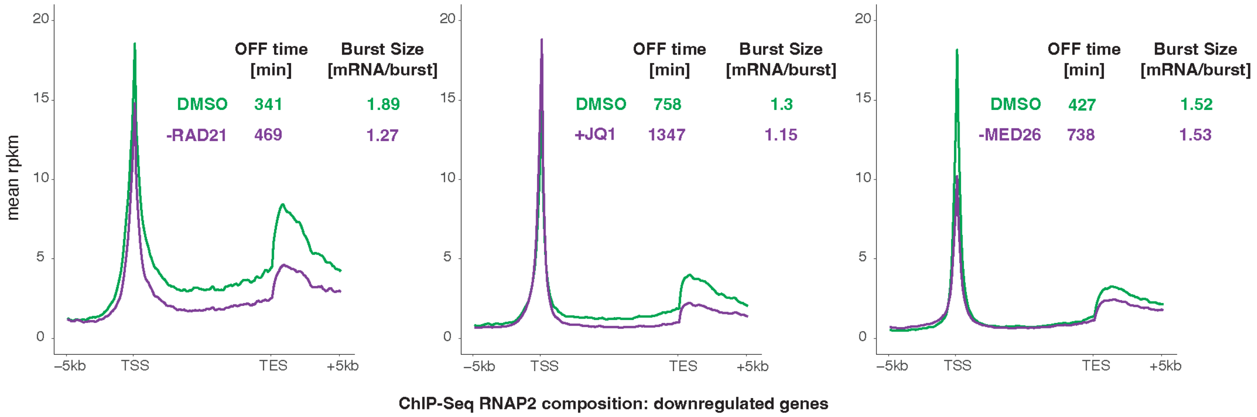

Fig. S13.

(related to Fig. 5). RAD21, BRD4 (JQ1 treatment) and MED26 perturbations have distinct impacts on RNAP2 binding and bursting parameters. RNA Pol II (RNAP2) binding composites (based on ChIP-Seq) at downregulated genes upon cohesin loss (*n = 171 genes*), JQ1 treatment (*n = 425 genes*) and MED26 loss (*n = 475 genes*), along with inferred median OFF duration and burst size in control (DMSO) and upon perturbation in genes from Fig. 4B-C.

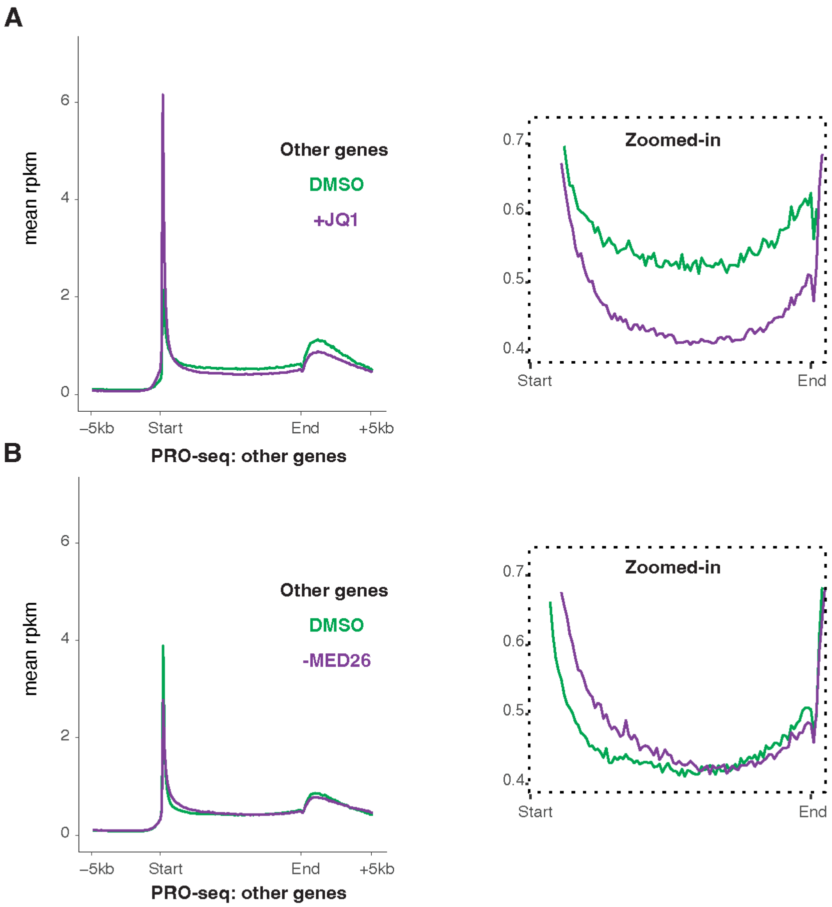

Fig. S14.

Other genes (other from those downregulated) exhibit different PRO-seq signals upon BRD4 (JQ1 treatment) and MED26 perturbations around the gene body. (A) PRO-seq composites of transcriptionally engaged RNA polymerases upon JQ1 treatment at genes other than significantly downregulated ones *(n = 6,248)*, and zoomed-in view around the gene body *(in green: DMSO and in magenta: +JQ1)*. **(B)** PRO-seq composites of transcriptionally engaged RNA polymerases upon MED26 perturbations at genes other than significantly downregulated ones *(n = 6,799)*, and zoomed-in view around the gene body *(in green: DMSO and in magenta: -MED26)*.

Data S1. (separate file)

Supplementary Table 1: 1, 2, 3- state model winners in the steady state HCT-116 cells.

Data S2. (separate file)

Supplementary Table 2: Cell numbers: smRNA-FISH and scRNA-Seq mRNA (Fig. 1B and Fig. 4D).

Data S3. (separate file)

Supplementary Table 3: smRNA-FISH and scRNA-Seq mRNA counts (Fig. 1B and Fig. 4D).

Data S4. (separate file)

Supplementary Table 4: list of differentially expressed genes upon RAD21 loss (Fig. 5A).

Data S5. (separate file)

Supplementary Table 5: list of differentially expressed genes upon JQ1 treatment (Fig. 5A).

Data S6. (separate file)

Supplementary Table 6: list of differentially expressed genes upon MED26 loss (Fig. 5A).

Data S7. (separate file)

Supplementary Table 7: list of downregulated genes (Fig. 5A).

Data S8. (separate file)

Supplementary Table 8: list of smRNA-FISH probes.
